# Alterations in cellular expression in EBV infected epithelial cell lines and tumors

**DOI:** 10.1371/journal.ppat.1008071

**Published:** 2019-10-04

**Authors:** Rachel Hood Edwards, Robert Dekroon, Nancy Raab-Traub

**Affiliations:** 1 Lineberger Comprehensive Cancer Center University of North Carolina at Chapel Hill Chapel Hill, North Carolina, United States of America; 2 Department of Microbiology & Immunology University of North Carolina at Chapel Hill Chapel Hill, North Carolina, United States of America; University of Wisconsin-Madison School of Medicine and Public Health, UNITED STATES

## Abstract

The Epstein Barr virus (EBV) is linked to the development of two major epithelial malignancies, gastric carcinoma and nasopharyngeal carcinoma. This study evaluates the effects of EBV on cellular expression in a gastric epithelial cell line infected with or without EBV and a nasopharyngeal carcinoma cell line containing EBV. The cells were grown *in vitro* and as tumors *in vivo*. The effects on cellular expression were determined using both 2D DIGE proteomics and high throughput RNA sequencing. The data identify multiple pathways that were uniquely activated *in vitro*. RNA sequences mapping to the mouse genome were identified in both the EBV positive and negative tumor samples *in vivo*, although, differences between the EBV positive and negative cells were not apparent. However, the tumors appeared to be grossly distinct. The majority of the identified canonical pathways based on two fold changes in expression had decreased activity within the tumors *in vivo*. Identification of the predicted upstream regulating factors revealed that *in vitro* the regulating factors were primarily protein transcriptional regulators. In contrast, *in vivo* the predicted regulators were frequently noncoding RNAs. Hierarchical clustering distinguished the cell lines and tumors, the EBV positive tumors from the EBV negative tumors, and the NPC tumors from the gastric tumors and cell lines. The delineating genes were changed greater than 4 fold and were frequently regulated by protein transcription factors. These data suggest that EBV distinctly affects cellular expression in gastric tumors and NPC and that growth *in vivo* requires activation of fewer cellular signaling pathways. It is likely that the broad changes in cellular expression that occur at low levels are controlled by regulatory viral and cellular RNAs while major changes are affected by induced protein regulators.

## Author summary

This study analyzes the effects of the Epstein Barr virus on cellular RNA expression in epithelial cells that were grown in culture dishes or were inoculated into immunodeficient mice to form tumors. The presence of EBV induced many changes in expression and affected the appearance of the tumors which were very bloody with EBV and solid and white without EBV. Multiple cellular pathways that were activated during growth in culture were decreased when grown as tumors. Many of the cellular genes that were affected and were changed approximately two fold were predicted to be regulated by noncoding RNAs. The clustering expression analysis identified greater changes in expression and these changes distinguished tumors from the cell lines, the gastric cells from the nasopharyngeal cancer cells, and those that contained EBV from the EBV negative cells. These findings indicate that EBV alters cell growth through major effects on cellular RNA transcription and that these changes can be induced by both noncoding RNAs and cellular transcription factors.

## Introduction

The Epstein-Barr Virus (EBV) is a major opportunistic pathogen that induces multiple malignancies including Burkitt (BL), Hodgkin (HL), diffuse large B cell lymphoma (DLBCL), primary effusion lymphomas (PEL) in coinfections with Kaposi sarcoma virus (KSHV), central nervous system lymphoma (CNS) and several carcinomas including gastric carcinoma (GC), and nasopharyngeal carcinoma (NPC) [1]. EBV readily infects B-lymphocytes and induces continuous growth *in vitro*, however, infection of epithelial cells is much more difficult and cell lines and xenografts have been difficult to establish from NPC and GC [1]. Several well studied NPC xenografts, C15 and C17 can be easily passaged in immunodeficient mice but cannot be cultivated *in vitro*, while the C666 cell line is a rare example of an NPC that can be cultivated *in vitro* and retains the EBV genome [2–4]. The AGS cell line was established from an EBV negative adenoma and is one of the few epithelial cell lines that can be infected with EBV [5]. Different patterns of viral gene expression are characteristic of the distinct malignancies and are also observed in cell lines and xenografts [1, 6]. B-lymphocytes infected *in vitro* express six nuclear antigens (EBNA), two membrane proteins, the abundant pol 3 EBER transcripts, the BHRF1 miRNAs, and low levels of the non-coding BART RNAs [7]. This pattern of expression is termed Type III latency. The C15 retains a pattern of EBV expression much like NPC and is considered Type II latency with expression of EBNA1, latent membrane protein 1 (LMP1), latent membrane protein 2 (LMP2), the non-coding Pol III EBER RNAs, and the non-coding BART RNAs (BamHI A rightward transcripts) [6]. EBV infection of AGS cells mirrors EBV positive gastric cancer and consistently results in Type 1 latency with expression of EBNA1 and the BART RNAs [8, 9]. The BART RNAs are templates for 44 virally encoded miRNAs while the spliced polyadenylated BART RNAs remain nuclear and apparently function as long noncoding (lnc) RNAs with consistent effects on cell expression [10, 11]. Additionally, EBV infection of the AGS gastric cell line induces anchorage independent growth with major effects on cellular mRNA expression, despite the highly restricted Type I pattern of expression [8]. Expression of a single form of the spliced BART RNAs had considerable effects on cellular expression indicative of lnc function [11, 12]. A recent study used orthotopic injection of the C666 cell line into the nasopharynx and also nasopharyngeal injection of AGS-EBV cells to evaluate tumor growth and metastasis [13]. The study indicated that expression of the BART miRNAs increased *in vivo* and that they were major effectors of changes in growth *in vivo*. This study also reported that AGS-EBV cells did not form tumors subcutaneously but only when inoculated into the nasopharynx, although the original establishment of the AGS cells reported tumor growth subcutaneously [14].

As the BART RNAs are the most abundant transcripts in both NPC and GC and are thought to primarily affect growth through effects on cellular expression, the parental AGS cell line, AGS-EBV cell line, and the C666 NPC cell line were inoculated both subcutaneously (sc) and intraperitoneally (ip) into immmunodeficient mice. Additionally, the NPC xenografts, C15 and C17, were inoculated (sc) and included for comparison. All cell lines with or without EBV formed tumors both sc and ip although the AGS tumors with EBV were visually quite distinct from the EBV negative AGS tumors. Proteomic and RNA Seq analysis indicated that cellular expression was very different in gastric cells compared to NPC and that cellular expression *in vitro* was very different from that observed during tumor formation *in vivo*. Interestingly there were surprising differences in the predicted upstream regulators in both the EBV positive and EBV negative lines and tumors.

## Results

### EBV infection minimally affects growth rate *in vivo*

**Fig 1:**
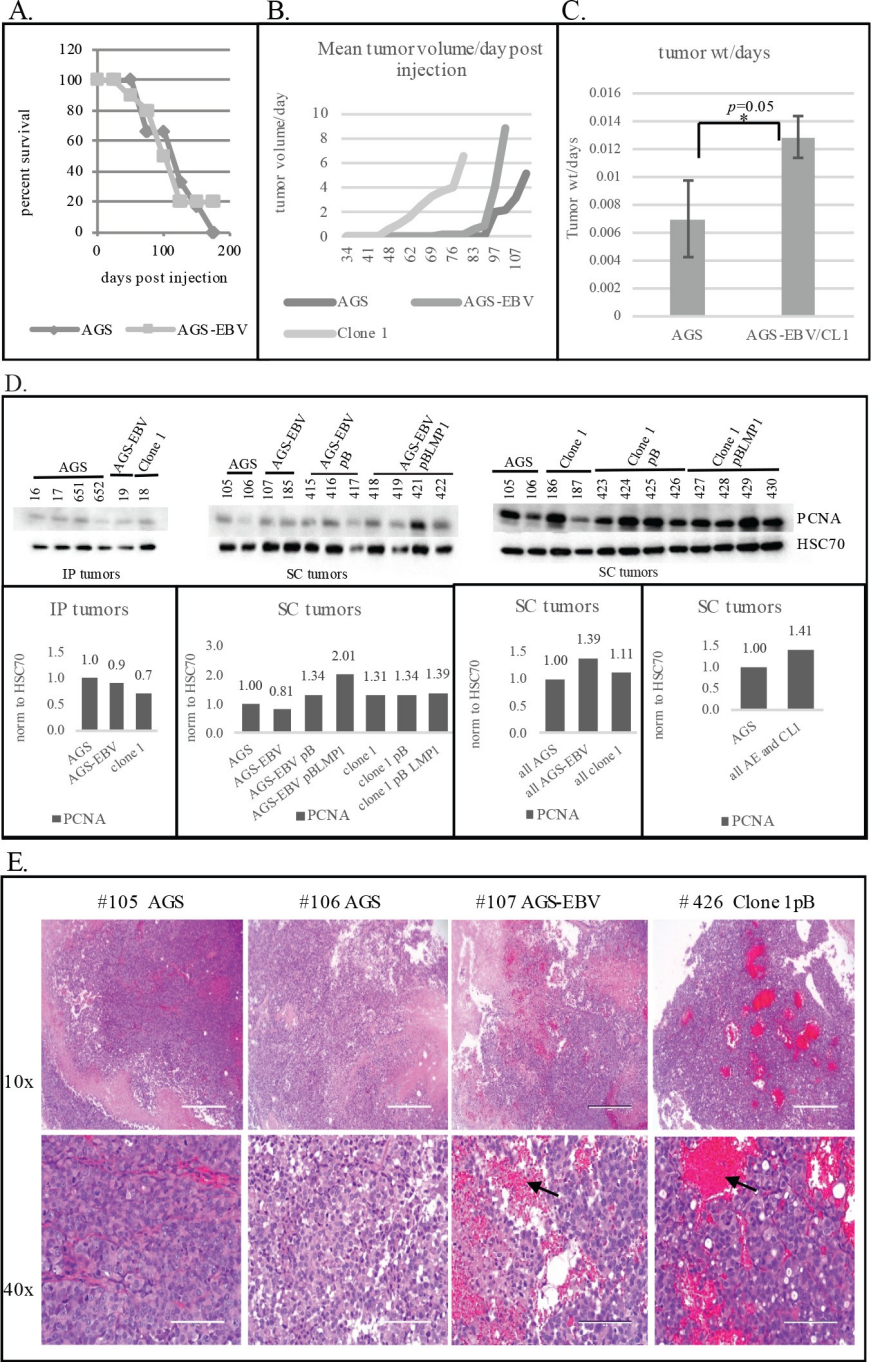
Survival, tumor growth, and proliferation rates of EBV^-^ AGS tumors and EBV^+^ AGS-EBV/Clone 1 tumors that develop in mice. (A) Kaplan-Meier survival plot of AGS injected mice and the AGS-EBV/Clone 1 injected mice as a function of days post injection subcutaneously (SC) or intraperitoneally (IP). (B) Average growth rate of the tumors post subcutaneous injection (SC) of the cell lines measured in tumor volume and days post injection. Graph of mean tumor volume at days post subcutaneous injection for each of the three cell lines, AGS; AGS-EBV and pB derivatives; and Clone1and pB derivatives. (C) Graph of mean tumor weight at harvest/days post injection at harvest of the AGS tumors (IP and SC) and the AGS-EBV and Clone1 and pB derivatives (IP and SC). (D) Western blot detection of PCNA in the IP and SC tumors that develop following injection of the AGS, AGS-EBV, and Clone1 cell lines and pB derivatives and quantification of the PCNA normalized to HSC70 as a loading control using the Image Lab software (Biorad). (E) Haemotoxylin and eosin staining of representative AGS, AGS-EBV, and Clone 1 tumors. Arrows denote presence of red blood cells.

The AGS, AGS-EBV, and C666 cell lines have previously been described [3, 5, 14]. Additionally, as LMP1 is a potent EBV encoded oncogene, derivatives of the AGS-EBV cell lines were prepared that contained the pBABE expression vector with or without LMP1 coding sequences. Although LMP1 transcription could be detected at low levels, LMP1 protein was not detected and likely did not affect growth. Clone 1 is a clone of AGS-EBV that has been maintained separately but is not obviously distinct [8]. However, these cell lines and tumors were also analyzed for increased statistical power. The mice were inoculated either subcutaneously (sc) or intraperitoneally (ip) (Table 1). All animals were sacrificed according to IACUC regulations based on physical appearance or distress. The tumors were identified and harvested (Table 1). Surprisingly, the tumors were grossly distinct at time of sacrifice with the AGS cells forming solid white tumors sc while the AGS-EBV tumors were soft with excessive amounts of blood. Histopathologic examination indicated that the AGS tumors had extensive necrosis (Table 1) and had evidence of differentiation in the IP injections. The AGS-EBV tumors also had some necrosis with considerable hemorrhage. The time to sacrifice did not obviously differ between the AGS parental cells and the collective AGS-EBV cells (Table 1, Fig 1A) nor did the insertion of the pBABE expression vector to express LMP1 or vector control affect growth (Table 1). The apparent growth rate determined by tumor volume at time of harvest was also variable with clone 1 necessitating sacrifice slightly earlier (Fig 1B). However, assessing tumor weight and combining all of the EBV positive tumors with or without exogenous vector in comparison with the AGS tumors revealed a slightly enhanced growth of the EBV positive tumors with borderline significance (P = 0.05) (Fig 1C). To further investigate any growth stimulating effects induced by EBV, tumor lysates were analyzed by immune blotting for proliferating cell nuclear antigen (PCNA) as a marker for DNA synthesis [15]. The detection was quantified, normalized to the cellular marker HSC70, and presented graphically relative to AGS tumors (Fig 1D). The PCNA detection was also variable and did not reveal statistically significant differences. This is in agreement with the histologic determination of mitotic index which was similar in the AGS and AGS-EBV tumors but elevated in the NPC C666 tumors (Table 1). Haemotoxylin and eosin staining of the tumor tissues confirmed the presence of abundant red blood cells in the EBV^+^ gastric tumors as compared to the EBV^-^ gastric tumors (Fig 1E).

**Table 1:** Tumor samples and histology.

Cell line Injected	Route of injection	Number of tumors	Average time to harvest (days)	Average tumor weight (grams)	Average mitotic index*	Histology Comments
AGS	IP	4	83	0.72	1.75 to 6	Small dense nuclei, trying to form ducts, necrosis
AGS-EBV	IP	1	56	0.93	3 to 7	Hemorrhage and necrosis
Clone 1	IP	1	48	1.45	4 to 11	Masses of polygonal cell, large nuclei, more vascular, hemorrhage and necrosis
C666.1	IP	4	44	2.52	13 to 29	Large cells and nucleoli, no necrosis or hemorrhage
AGS	SC	2	145	0.49	3 to 7	Masses of polygonal cells, hemorrhage and massive necrosis
AGS-EBV	SC	2	102	1.45	3 to 10	Hemorrhage and necrosis
AGS-EBVpB	SC	4	133	1.07	2.3 to 7.3	Very vascular, hemorrhage and necrosis, cuboidal to columnar cells sometimes forming variable sized glandular structures
AGS-EBV pBLMP1	SC	3	151	1.70	2.3 to 7.7	Very vascular, hemorrhage and necrosis, cuboidal to columnar cells sometimes forming variable sized glandular structures
Clone 1	SC	1	86	0.44	**	necrosis
Clone 1pB	SC	4	82	0.88	5.25 to 13	Solid sheets of tumor cells, necrosis, hemorrhage, vascular pools
Clone 1 pBLMP1	SC	4	82	1.39	2.3 to 11.3	Massive necrosis, pools of blood, hemorrhage

*number of mitosis per 40x magnification field

**sample too necrotic

### Distinct cellular protein expression in cell lines and tumors

**Fig 2:**
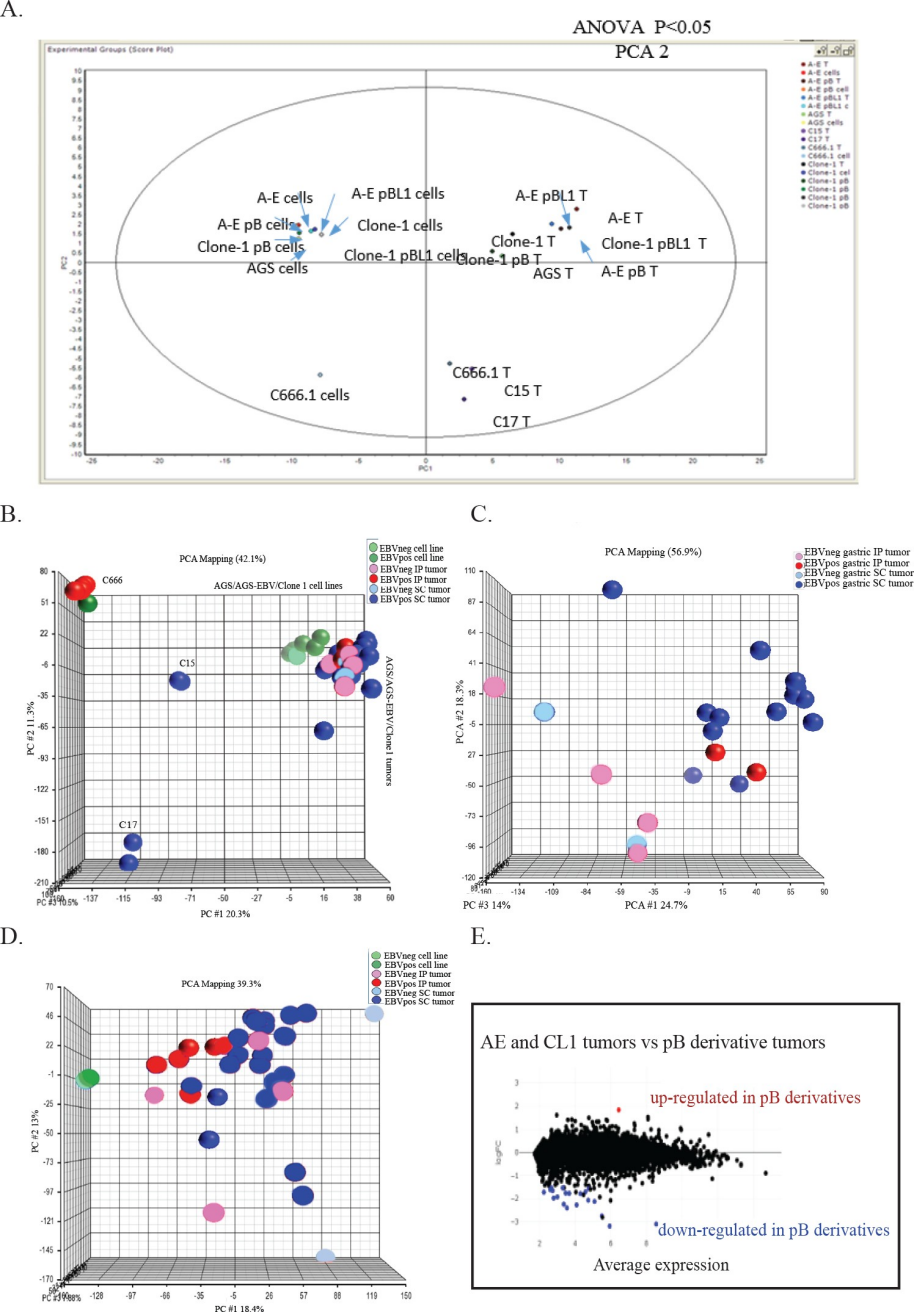
Principal component analysis based on 2D DIGE and RNA Seq analysis of the cell lines and tumors. (A) Principal component analysis of variation between proteins detected by 2D DIGE from the AGS, AGS-EBV, Clone1, pB derivatives, C666.1, and xenografts C15 and C17 cell lines and tumors. T represents tumors. (B) Principal component analysis based on variation between all expressed human transcripts from the AGS, AGS-EBV, Clone1, pB derivatives, C666.1, and xenografts C15 and C17 cell lines and tumors. (C) Principal component analysis based on variation between all expressed human transcripts from the AGS, AGS-EBV, Clone1, and pB derivatives gastric tumors. (D) Principal component analysis based on variation between all expressed mouse transcripts from the AGS, AGS-EBV, Clone1, and pB derivatives, C666.1, and xenografts C15 and C17 tumors. (E) Volcano plot of human gene expression of the pBabe derivative tumors vs the original cell line tumors. The few blue dots denote the significantly downregulated genes in the pBabe derivative tumors compared to the original cell line tumors and the red dots denote the significantly up regulated genes in the pBabe derivative tumors compared to the original cell line tumors.

We have previously shown that the BART RNAs including miRNAs and spliced RNAs are the most abundantly expressed RNAs in AGS-EBV cells [9]. As miRNAs in part target protein abundance, differences in protein expression were identified using 2-D difference gel electrophoresis. Analysis using Decyder software identified 1812 spots that matched across all samples. Further analysis using Principal Component Analysis (PCA) revealed that PCA1 separated the tumors from the cell lines representing at least 60.5% of the variance between cell lines and tumors of both AGS and AGS-EBV and the C666 cell line compared with C666 tumors (Fig 2A). This could reflect the presence of abundant mouse proteins contained within the mouse tumors grown *in vivo* in addition to growth *in vitro* compared to growth *in vivo*. Additionally, PCA2 completely distinguished AGS cell lines and tumors from the NPC C666 cell line and tumors (Fig 2A). The identification of the specific proteins within the distinguishing spots requires considerable material and the resulting identification is frequently obscured by abundant contaminating proteins. These data were primarily obtained to reveal global similarities and differences. To determine more specific differences, the tumors were analyzed further using RNA sequence analysis.

#### RNA sequence analysis distinguishes cell lines from tumors and NPC from AGS by principal component analysis

To more accurately identify effects on cellular expression, cell lines and all tumors including the NPC C15 and C17 tumors were analyzed by high throughput sequencing of polyadenylated selected RNA. The specific tumors, number of reads, aligned pairs, and mapped reads to human and mouse genomes are presented in Table 2. The number of reads mapping to the mouse genome (mm9) were quite variable and not obviously distinct between the sc or ip injections nor between the EBV positive and negative tumors (Table 2). Thus the distinct appearance of the EBV tumors with hemorrhage and increased blood did not represent invading mouse vessels. Interestingly, the percentage of reads mapping to the EBV genome decreased in the AGS-EBV tumors but not in the C666 NPC tumors (Table 2). There were very low levels of EBV expression in the C17 tumor as has been previously reported.

**Table 2:** TOPHAT summaries of reads to genomes.

Sample	Tumor and route	reads	Mapped reads to hg19	%	Aligned pairs	Concordant pair alignment rate	Mapped reads to mm9	%	Aligned pairs	Concordant pair alignment rate	Mapped reads to EBV (Akata)	%	Aligned pairs	Concordant pair alignment rate
AGS		25120188	23234918	92.1	21366251	84.2	260128	1.0	99700	0.4				
AGS	16(IP)	28216896	24374849	86.4	22551327	79.2	2025183	7.9	1757384	6.2				
AGS	17(IP)	26507596	23108678	87.2	21308764	79.7	1929337	7.3	16556132	6.2				
AGS	651(IP)	22396427	20218305	90.3	18932316	83.9	1069744	4.8	893020	3.9				
AGS	652(IP)	23802126	14723414	61.9	13575398	56.5	7676486	32.3	6800494	28.3				
AGS	105(SC)	22979420	19810978	86.2	18453532	79.6	1867618	8,1	1616988	6.9				
AGS	106(SC)	22280936	16079045	72.2	14908180	66.3	4969212	22.3	4555205	20.3				
AGS-EBV		63677984	61050483	95.9	55912111	86.5					222169	0.3	215243	0.3
AE	19(IP)	115554194	105020890	90.9	95950977	81.9	10910052	9.4	8382055	7.1	124070	0.1	119648	0.1
AE	107(SC)	95288761	83594814	86.7	76687846	79.5	11839257	12.3	9682229	10	46259	0.05	44639	0.05
AE	185(SC)	78863727	55865269	70.8	51001626	63.9	22673505	28.5	20124781	25.1	104755	0.1	100892	0.1
AGS-EBVpB		76574348	72369043	93.5	66446568	85.7					406864	0.5	394791	0.5
AEpB	415(SC)	71168887	58963301	81.9	54324259	75.5	11627145	16.2	9895911	13.7	87308	0.1	83400	0.1
AEpB	416(SC)	71226393	62539488	86.8	57492388	79.8	8195312	11.4	6660533	9.2	90672	0.1	87286	0.1
AEpB	417(SC)	70021038	53142325	75.0	48903732	69.1	15968270	22.6	14065701	19.8	144944	0.2	139638	0.2
AEpB	418(SC)	70833636	60015346	83.8	55335252	77.3	9979711	14.0	8434505	11.7	140395	0.2	135882	0.2
AGS-EBV pBLMP1		115047351	105960671	91.2	96953744	83.2	2659357	2.3	115487	0.7	471872	0.4	456441	0.4
AEL1	419(SC)	76758409	59412143	76.2	54335219	70.1	16625202	21.4	14431891	18.6	136432	0.2	130460	0.2
AEL1	421(SC)	78458488	66713274	84.1	61798325	78.0	11130082	14.1	9379617	11.8	150847	0.2	144873	0.2
AEL1	422(SC)	65292002	50066480	75.8	46191381	70.1	14626619	22.2	12914443	19.6	95393	0.1	91901	0.1
Clone1		103048645	95658583	91.9	86967029	83.1	2560564	2.5	756224	0.7	1533526	1.5	1486923	1.4
CL1	18(1P)	78017990	59833811	75.4	54138969	68.5	8557653	10.8	6900037	8.7	71137	0.1	67188	0.1
CL1	186(SC)	65221956	55893900	84.7	51090533	77.4	8935152	13.6	7365860	11.1	87797	0.1	84447	0.1
CL1	187(SC)	110876987	79666811	71.0	72183118	64.2	30720027	27.5	26950715	23.9	161116	0.1	155668	0.1
Clone1pB		82392572	74287997	89.2	67702984	80.9	1930323	2.4	515442	0.6	1381245	1.7	1340724	1.6
CL1pB	423(SC)	88917128	73074785	81.3	67189918	74.7	15595795	17.4	13405332	14.9	76208	0.1	73506	0.1
CL1pB	424(SC)	56750359	47420631	82.7	42716906	74.1	8456178	14.9	7059000	12.2	191995	0.3	187045	0.3
CL1pB	425(SC)	69238606	59374586	84.8	54491966	77.8	8809215	12.6	7316106	10.4	176411	0.3	170509	0.2
CL1pB	426(SC)	61840262	53720905	86.0	49830877	79.9	7353930	11.8	6206115	9.9	52634	0.1	51146	0.1
Clone1 pB LMP1		64447535	58888159	90.3	53121229	81.1					346401	0.5	334768	0.5
C1L1	427(SC)	83151529	70175691	83.4	64199105	76.3	12311478	14.7	10400537	12.3	55651	0.1	53723	0.1
C1L1	428(SC)	69379810	60335980	86.2	55605976	79.2	8262995	11.8	6818448	9.7	120715	0.2	116787	0.2
C1L1	429(SC)	70559921	61249718	85.6	55952381	78.4	9007046	12.6	7393909	10.3	89335	0.1	85940	0.1
C1L1	430(SC)	71157444	58643359	81.3	53838297	74.9	12037784	16.7	10306923	14.3	55117	0.1	53085	0.1
C666.1		74627397	72620949	96.3	65856734	86.7	2006842	2.7	710300	0.9	18744	0.03	17851	0.03
C666.1	22(IP)	69024264	62038997	88.9	56218304	80.0	6915798	10.0	5445516	7.8	27651	0.04	26489	0.04
C666.1	23(IP)	80733054	69690794	85.4	63089106	76.8	10842027	13.4	8995581	11.0	24602	1.0	23525	1.0
C666.1	103(IP)	56528824	51958245	90.9	46831925	81.3	4395389	7.8	3244220	5.6	16319	0.03	15640	0.03
C666.1	104(IP)	73297880	66607704	89.9	59772990	80.0	6075264	8.3	4656778	6.2	36845	0.05	35264	0.05
C15	2005	80772880	57955965	71.0	51735775	62.6	22715151	27.9	19174495	22.6	14842	0.02	13039	0.02
C15	2014	73537039	52267135	70.4	46737781	62.1	20994674	28.3	17748882	22.9	23042	0.03	20688	0.03
C17	1997	84853841	72291590	84.3	64454424	74.4	12774604	15.0	10442092	12.0	495	0.001	454	0.001
C17	2005	51557626	43374069	83.1	38171939	72.2	8396243	16.2	6855026	12.9	202	0.001	185	0.001

Aligned reads were mapped to specific human Refseq genes using the Partek Genomics Suite. Partek and Biojupies were used to identify differentially expressed genes, generate hierarchical clusters, and perform principal component analysis (PCA). PCA in comparison with the human genome indicated that PCA1 determined that approximately 20% of the distinction was between NPC and the AGS cell lines and the tumors. Each NPC consistently clustered distinctly (Fig 2B). The C666 cell line clustered completely with the C666 tumors. This is interesting as C666 is a rare NPC that can grow as a cell line and these data confirm their similarity and identity [3, 4]. The AGS and AGS-EBV cell lines clustered together but were distinguishable from the tumors (Fig 2B). PCA analysis did not distinguish the AGS tumors from the AGS-EBV tumors (Fig 2B) nor whether the injection was sc or ip (Fig 2B). However, comparison of just the AGS tumors with or without EBV delineated the two groups (Fig 2C). Analysis of the differential expression of mouse genes did not reveal any clear differences between AGS or AGS-EBV tumors using principal component analysis (Fig 2D). This suggests that the biologic difference in the amount of blood and hemorrhage reflects distinct growth properties of the AGS-EBV tumors from the AGS tumors that are likely due to differences in cellular expression within the AGS-EBV tumor cells and that the increased hemorrhage does not reflect infiltrating mouse tissue. To confirm that the addition of the pBabe vector did not significantly change the human or mouse gene expression in the AGS-EBV positive tumors as compared to the original cell line AGS-EBV tumors, volcano plots were generated comparing gene expression in the tumors, and very few genes were significantly changed (Fig 2E).

#### Canonical pathway analysis of tumors and cell lines

**Fig 3:**
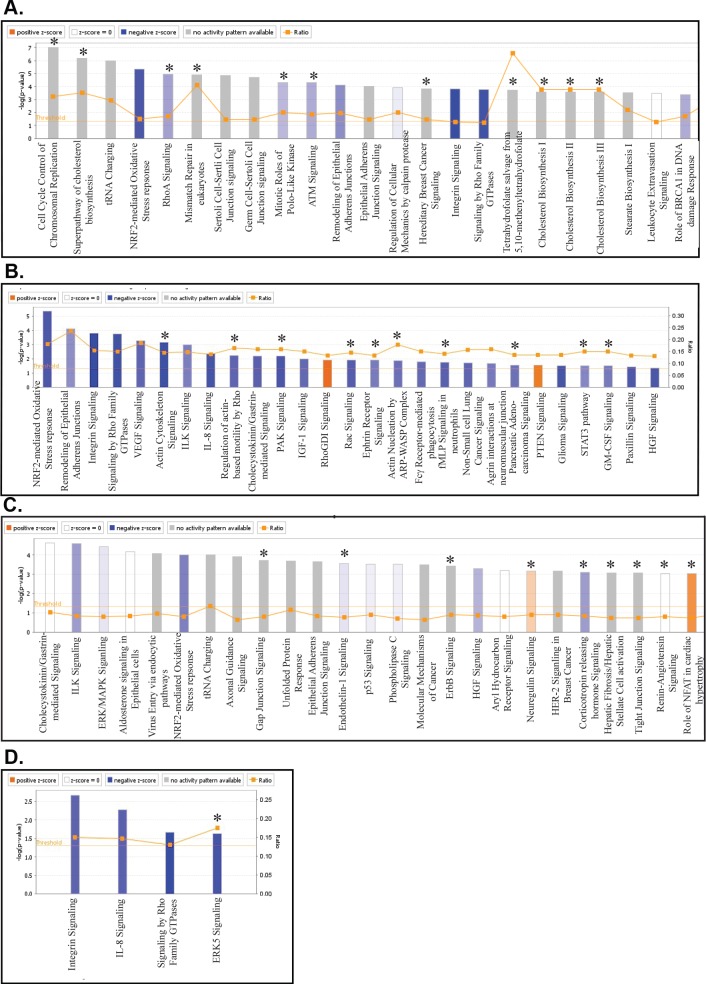
Canonical pathways associated with human genes with 2-fold expression changes by RNA Seq in gastric tumors when compared to the gastric cell lines. (A) Top canonical pathways associated with human genes with 2 fold expression change by RNA seq in the AGS tumors compared to AGS cell line. Astericks (*) denote pathways unique to the comparison of AGS tumor to their cell line. (B) Significant canonical pathways (absolute z-score ≥2) associated with AGS tumors compared to AGS cell line. The height of the bars reflects the p value, and the orange boxes reflect the ratio of the number of genes in the data set represented in the pathway. Astericks (*) denote pathways unique to the comparison of AGS tumor to their cell line. (C). Top canonical pathways associated with human genes with 2-fold expression change by RNA seq in the AGS-EBV tumors when compared to the AGS-EBV and pB derivative cell lines. Astericks (*) denote pathways unique to the comparison of AGS-EBV tumors to their cell lines. (D) Significant canonical pathways (absolute z-score ≥2) associated with AGS-EBV tumors compared to AGS-EBV and pB derivative cell lines. The height of the bars reflects the p value, and the orange boxes reflect the ratio of the number of genes in the data set represented in the pathway. Astericks (*) denote pathways unique to the comparison of AGS-EBV tumors to their cell lines.

Genes altered two fold by growth *in vivo* or *in vitro* were sorted into categories based on the canonical pathways using IPA software. The categories that were highly enriched in the data sets, with the size of each bar corresponding to the log of the *P* value of enrichment, are presented graphically. The orange boxes indicate the relative number of genes in the data set within that pathway indicated by the ratio at the right end of the graphs in Fig 3B and 3D. The orange line or threshold marks the p value of 0.05. Red bars indicate that the function is predicted to be activated, and blue bars indicate that the function is predicted to be inhibited based on the pattern of expression of these genes. Grey bars indicate that the function was predicted to be neither activated nor inhibited based on the data. Comparison of the AGS tumors to the AGS cell lines identified multiple pathways that had significant enrichment but could not be predicted to be activated or inhibited but were apparently affected indicating that multiple genes that contribute to these pathways differed in the tumors and cell lines (Fig 3A). Pathways uniquely affected in the AGS tumor or the AGS-EBV tumors are indicated with astericks. Identification of only those pathways that had a significant predicted change evidenced by a z score change greater than 2 revealed that the majority of pathways were decreased in the tumors in comparison with the cell line (Fig 3B). These included pathways likely involved in growing in adherent condition *in vitro* including NRF2 oxidative stress response, remodeling of adherans junctions, integrin signaling, signaling by rho family members, and interleukin signaling (ILK). Several pathways including actin cytoskeleton signaling, Rac signaling, Pac signaling, and Stat3 pathway were predicted to be uniquely downregulated in the AGS tumors compared to their cell line but not in the AGS-EBV tumors. Only Rho GDI signaling and PTEN signaling were activated and unique to the AGS tumors (Fig 3B). Comparison of the AGS-EBV tumors to the AGS-EBV cell lines also identified multiple pathways in the data set without prediction of activation and also identified several of the same pathways identified in the AGS tumors, including ILK signaling, ERK/MAPK signaling, and epithelial adherans junction signaling. However, fewer pathways were predicted to be inhibited (Fig 3C and 3D). The integrin, IL-8, and Rho GTPase signaling pathways were predicted to be inhibited with significant z scores in both the EBV positive and negative tumors as compared to their cell lines, but the ERK5 signaling pathway was specifically inhibited in the EBV positive tumors (Fig 3D). Analysis of canonical pathways affected in AGS-EBV tumors in comparison with AGS tumors had few pathways significantly affected (S1A Fig) with decreased activation of intrinsic prothrombin activation and phospholipases in the EBV positive AGS tumors (S1B Fig). Interestingly several metabolic pathways including glutamine degradation and glucaronate degradation had multiple genes affected although neither activation nor inactivation was predicted (S1A Fig).

In agreement with PCA, the analysis of the predicted canonical pathways from the gene expression in the C666 tumors as compared to the cell line did not identify any significant differences except in the predicted inhibition of calcium signaling in the tumors (S2 Fig).

As expected, multiples pathways were significantly and uniquely changed when comparing the NPC tumors to the EBV negative and EBV positive gastric tumors. Three pathways involved in cholesterol biosynthesis were activated in the NPC tumors as compared to the gastric tumors while integrin signaling was predicted to be inhibited (S3 Fig). 14-3-3 signaling and phospholipase signaling were apparently more important in the AGS tumors, while SAPK/JNK signaling was more important in the AGS-EBV tumors (S3A and S3B Fig).

#### Predicted upstream regulators are altered in tumors and indicate increased regulation by miRNAs

Potential upstream regulators of these changes in expression with significant p-values were also identified using IPA analysis. The vast majority, 23 in all, of the predicted activators of expression in the EBV negative AGS tumors were identified as miRNAs (Table 3). While the RNA Seq data of the tumors does not identify the mature miRNA, their expression in the cell lines was identified in previous studies [9]. Several of these miRs were expressed in the AGS cell line. Several of the miRNAs predicted to be upstream regulators and activated in the tumors had a seed sequence similar to specific BART miRNAs (Table 3). Only 3 of the activated regulators which also had significant increase in expression were proteins including TCF3, COL18A1, and HNF1A. These findings suggest that regulation of growth *in vivo* by protein regulators is decreased and that significant regulation is mediated by noncoding RNAs.

**Table 3:** Upstream regulators predicted for AGS tumor vs AGS cell line.

Upstream Regulator*	Expr Fold Change**	Molecule Type***	Predicted Activation State	Activation z-score	p-value of overlap	# of target molecules in dataset with expression consistent with activation or inhibition
MYC	-2.69	transcription regulator	Inhibited	-5.027	4.16E-16	95/167
**E2F3**	-1.58	transcription regulator	Inhibited	-3.376	3.65E-13	24/44
E2F2	-2.07	transcription regulator	Inhibited	-2.673	4.81E-13	12/27
**CCND1**	-3.58	transcription regulator	Inhibited	-4.075	1.35E-11	30/58
**ATF4**	-1.75	transcription regulator	Inhibited	-4.656	2.24E-10	32/39
TCF7L2	-1.61	transcription regulator	Inhibited	-3.259	7.47E-09	40/59
**KRAS**	-1.99	enzyme	Inhibited	-3.33	9.36E-07	31/70
**EDN1**	-9.45	cytokine	Inhibited	-4.065	8.33E-06	27/36
**CSF2**	turned off in tumor	cytokine	Inhibited	-5.457	1.13E-05	49/65
**SREBF2**	-2.82	transcription regulator	Inhibited	-2.482	2.53E-05	14/18
**CCNE1**	-2.29	transcription regulator	Inhibited	-2.121	3.74E-05	7/9
MKL1	-1.82	transcription regulator	Inhibited	-3.223	4.03E-05	15/26
**FOXO1**	-4.25	transcription regulator	Inhibited	-2.804	5.97E-05	29/51
**ETS1**	-6.58	transcription regulator	Inhibited	-3.061	9.77E-05	15/35
**EGFR**	-8.76	kinase	Inhibited	-3.798	0.000141	36/53
**AIF1**	-8.41	other	Inhibited	-2.236	0.00201	5/5
CREB1	-1.72	transcription regulator	Inhibited	-2.962	0.00318	36/65
**HIF1A**	-3.72	transcription regulator	Inhibited	-2.212	0.00501	25/48
**MAP2K1**	-2.21	kinase	Inhibited	-2.553	0.00541	14/25
MET	-3.78	kinase	Inhibited	-3.208	0.0071	13/15
EIF4E	-1.82	translation regulator	Inhibited	-3.299	0.0107	16/20
**KLF5**	-1.87	transcription regulator	Inhibited	-2.016	0.0136	8/10
DBI	-1.72	other	Inhibited	-2	0.0143	4/4
**MAP2K4**	-1.53	kinase	Inhibited	-2.595	0.0195	7/10
**CCNK**	-1.89	kinase	Inhibited	-2.376	0.0362	6/6
**PRKCE**	-3.15	kinase	Inhibited	-2.745	0.0376	10/11
**IL15**	turned off in tumor	cytokine	Inhibited	-2.288	0.0394	16/41
**let-7 (MIRLET7BHG)**	3.24	microrna	Activated	4.771	9.45E-10	33/37
miR-16-5p (miRs w/seed AGCAGCA)	exp in cell line	mature microrna BART-20-5p AGCAG**G**C	Activated	4.654	1.12E-07	36/41
miR-1-3p	not exp in cell line	mature microrna	Activated	3.413	1.96E-06	29/37
miR-124-3p		mature microrna	Activated	4.427	4.75E-05	35/41
miR-34a-5p	2.57	mature microrna	Activated	2.485	1.59E-05	12/15
miR-155-5p (miRs w/seed UAAUGCU)	not exp in cell line	mature microrna BART14 **A**AATGCT	Activated	3.899	1.7E-05	26/30
miR-21-5p	not exp in cell line	mature microrna	Activated	2.957	1.97E-05	14/16
miR-503-5p (miRs w/seed AGCAGCG)	not exp in cell line	mature microrna BART1-3p AGCA**C**CG BART2-3p AG**G**AGCG BART20-5p AGCAG**GC**	Activated	2.586	5.96E-05	7/7
**TCF3**	2.41	transcription regulator	Activated	3.285	0.000205	22/35
miR-30c-5p	exp in cell line	mature microrna	Activated	3.177	0.000265	18/21
**let-7a-5p**	3.24	mature microrna	Activated	3.577	0.000313	22/26
miR-291a-3p		mature microrna	Activated	4.217	0.000331	18/18
mir-122	not exp in cell line	microrna	Activated	3.765	0.000331	17/18
miR-145-5p	exp in cell line	mature microrna	Activated	2.021	0.000738	10/13
mir-21	highly exp in cell line	microrna	Activated	4.475	0.000781	32/37
mir-15	exp in cell line	microrna	Activated	2.255	0.00125	9/12
miR-199a-5p	not exp in cell line	mature microrna	Activated	2.244	0.00184	11/14
miR-133a-3p	not exp in cell line	mature microrna	Activated	2.596	0.00296	10/11
mir-34 (MIR34HG)	2.57	microrna	Activated	2.39	0.0036	9/10
COL18A1	11.59	other	Activated	3.333	0.0071	14/15
**HNF1A**	43.15	transcription regulator	Activated	2.88	0.00765	22/52
miR-30a-3p	exp in cell line	mature microrna	Activated	2.447	0.0102	6/6
miR-29b-3p (miRs w/seed AGCACCA)	exp in cell line	mature microrna BART1-3p AGCACC**G**	Activated	3.249	0.0135	11/11
miR-26a-5p	highly exp in cell line	mature microrna	Activated	2.388	0.0224	6/6
miR-92a-3p	highly exp in cell line	mature microrna	Activated	2.412	0.0265	6/6
miR-203a-3p	exp in cell line	mature microrna	Activated	2.236	0.033	5/5

*Bolded font denotes genes with fold change in expression with p value and FDR <0.05, regular font denotes fold change in expression with p<0.05

**EBV miR expression from [9]

***Bolded font denotes base change in EBV miR

Similarly, in the AGS-EBV tumors, many protein regulators were predicted to be inhibited (Table 4). Four protein regulators that were activated included ALDH2, COL18A1, TRIM24, and HDAC1. The activation of HDAC1 uniquely in the EBV positive tumors also suggests the importance of epigenetic regulation during tumor formation. Activated regulators also included 4 miRNAs with two that differed from those detected in the AGS tumors, miR-8 and miR-27a-3p. It is possible that EBV BART miRNAs or lnc RNAs may substitute for some of the activated miRNAs in the AGS tumors.

**Table 4:** Upstream regulators predicted for AGS-EBV tumors vs cell lines.

Upstream Regulator*	Expr Fold Change**	Molecule Type	Predicted Activation State	Activation z-score	p-value of overlap	# of target molecules in dataset with expression consistent with activation or inhibition
**ATF4**	-1.95	transcription regulator	Inhibited	-5.913	8.09E-32	47/63
**EGFR**	-5.84	kinase	Inhibited	-3.09	2.09E-13	41/67
**HIF1A**	-1.78	transcription regulator	Inhibited	-2.581	1.65E-12	39/75
**NUPR1**	-3.76	transcription regulator	Inhibited	-4.638	3.16E-12	67/91
**EDN1**	-2.76	cytokine	Inhibited	-3.873	5.5E-11	32/47
**SRF**	-1.53	transcription regulator	Inhibited	-3.017	2.41E-10	21/64
**DDIT3**	-3.32	transcription regulator	Inhibited	-2.374	4.0E-09	15/21
**EP300**	-1.33	transcription regulator	Inhibited	-2.052	6.99E-08	17/69
**MAP2K1**	-1.45	kinase	Inhibited	-2.976	1.1E-07	22/37
**EZH2**	-1.55	transcription regulator	Inhibited	-2.533	2.29E-07	23/52
**EGR1**	-18.54	transcription regulator	Inhibited	-2.071	2.36E-07	19/37
**NOTCH1**	-2.75	transcription regulator	Inhibited	-2.71	4.58E-07	26/43
**MAP2K1/2**	-1.4/-1.5	group	Inhibited	-3.516	1.02E-06	17/19
**MET**	-2.87	kinase	Inhibited	-2.429	2.24E-06	16/22
**IPMK**	-1.51	kinase	Inhibited	-3.111	4.49E-06	10/10
**FOXO1**	-1.55	transcription regulator	Inhibited	-2.082	6.13E-06	29/54
**TGFA**	-1.79	growth factor	Inhibited	-3.249	9.43E-06	15/20
**IRS1**	-2.58	enzyme	Inhibited	-2.088	1.02E-05	7/26
**YAP1**	-1.46	transcription regulator	Inhibited	-2.632	1.58E-05	9/19
**CREB1**	-1.57	transcription regulator	Inhibited	-3.564	1.65E-05	43/75
**RPS6KA5**	-5.14	kinase	Inhibited	-2.147	1.76E-05	7/8
**KLF5**	-1.79	transcription regulator	Inhibited	-2.094	2.72E-05	11/15
**IGF1R**	-1.50	transmembrane receptor	Inhibited	-3.25	4.37E-05	22/38
**PPRC1**	-1.44	transcription regulator	Inhibited	-3.046	5.9E-05	12/13
**TEAD1**	-1.61	transcription regulator	Inhibited	-2.157	6.93E-07	7/11
**F2RL1**	-2.42	g-protein coupled receptor	Inhibited	-2.011	0.000141	9/14
**PRKCE**	-4.49	kinase	Inhibited	-3.669	0.00027	15/16
**MAP2K4**	-1.15	kinase	Inhibited	-3.127	0.000637	10/13
**GAST**	-23.17	other	Inhibited	-2.509	0.000698	13/16
**CREM**	-1.46	transcription regulator	Inhibited	-2.091	0.000855	15/25
**ELF4**	-1.80	transcription regulator	Inhibited	-2.137	0.000999	7/9
**NFATC3**	-1.38	transcription regulator	Inhibited	-2.374	0.00107	8/13
**RHOB**	-1.51	enzyme	Inhibited	-2.2	0.00308	5/5
**Stat3-Stat3**	-1.31	complex	Inhibited	-2.415	0.0128	6/6
**CDKN1A**	-4.69	kinase	Inhibited	-2.799	0.0285	15/27
**CARM1**	-1.36	transcription regulator	Inhibited	-2.383	0.0375	6/7
**CHUK**	-1.45	kinase	Inhibited	-2.416	5.2E-07	23/40
**ALDH2**	1.76	enzyme	Activated	3.45	7.07E-08	12/12
**COL18A1**	2.07	other	Activated	3.703	2.17E-07	21/24
**TRIM24**	2.12	transcription regulator	Activated	2.496	0.0329	11/14
**HDAC1**	1.50	transcription regulator	Activated	2.324	3.74E-09	2050
miR-199a-5p (miRs w/seed CCAGUGU)	exp in cell line	mature microrna	Activated	2.712	5.42E-05	14/17
miR-30c-5p (miRs w/seed GUAAACA)	exp in cell line	mature microrna	Activated	2.584	9.86E-05	17/22
mir-8		microrna	Activated	2.555	0.000627	13/15
miR-27a-3p (miRs w/seed UCACAGU)	exp in cell line	mature microrna	Activated	2.607	0.0375	7/7

*Bolded font denotes genes with fold change in expression with p value and FDR <0.05, regular font denotes fold change with p<0.05

**miR expression from [9]

However, comparison of upstream regulators in EBV positive AGS tumors to EBV negative AGS tumors revealed effects on multiple pathways through both transcription factors and growth factors (Table 5). The predicted regulators were multiple genes previously shown to be affected by EBV including TNF, HIF1A, IL6, IRF7, and HNF1A which were predicted to be inhibited while CBX5, MAPK1,TP63, E2F3 were predicted to be activated (Table 5). These data are supported by the considerable numbers of genes detected in the data sets and the highly significant p-value of the overlap. It is intriguing that most of the predicted upstream regulators were inhibited in both EBV positive and negative tumors whereas miRNAs were primarily identified as activators of expression, particularly in the EBV negative AGS tumors (S1 Table).

**Table 5:** Upstream regulators predicted for AGS-EBV tumors vs AGS tumors.

Upstream Regulator*	Expr Fold Change	Molecule Type	Predicted Activation State	Activation z-score	p-value of overlap	# target molecules in dataset with expression consistent with activation or inhibition
TNF		cytokine	Inhibited	-2.804	6.54E-13	59/100
**NUPR1**	-1.92	transcription reg	Inhibited	-3.483	1.1E-06	35/53
IFNG		cytokine	Inhibited	-2.62	6.41E-06	49/72
CLDN7	-1.17	other	Inhibited	-2.173	0.000115	10/12
**HIF1A**	1.1	transcription reg	Inhibited	-2.000	0.000556	17/31
**TP53**	1.31	transcription reg	Inhibited	-2.015	0.000994	36/71
IL6		cytokine	Inhibited	-2.912	0.00941	40/78
**let-7 (MIRLET7BHG)**	-2.54	microRNA	Inhibited	-3.367	0.00103	20/23
**IL1 (A/B)**	-1.45/-1.27	group	Inhibited	-2.07	0.0248	18/29
IRF7	1.2	transcription reg	Inhibited	-2.09	0.0369	12/16
**HNF1A**	-1.93	transcription reg	Inhibited	-3.96	0.0393	23/35
**CBX5**	2.35	transcription reg	Activated	3.657	4.31E-10	23/27
**MAPK1**	1.54	kinase	Activated	2.723	6.39E-08	22/32
CCND1	-1.15	transcription reg	Activated	2.64	0.000144	19/41
TP63	1.5	transcription reg	Activated	2.883	0.00793	38/67
E2f **(E2F3)**	1.3	group	Activated	2.112	0.011	10/15
**TBX2**	1.4	transcription reg	Activated	2.35	0.0304	8/9
**EIF4G1**	1.64	translation reg	Activated	2.449	0.033	5/5

*Bolded font denotes genes with fold change in expression with p value and FDR <0.05, regular font denotes fold change in expression with p<0.05.

Identification of the Disease and Functions affected in the EBV positive SC tumors compared with the EBV negative SC tumors identified increased DNA replication, cell growth and proliferation, cancer, cellular development, and hematological system development and function. These functions are in keeping with the enhanced tumor growth. Additionally, the predicted activation of hematopoietic progenitor cells may likely reflect the highly bloody appearance of the EBV positive AGS tumors (Table 6).

**Table 6:** Disease and functions predicted for AGS-EBV SC tumors vs AGS SC tumors.

Categories	Diseases or Functions Annotation	p-Value	Predicted Activation State	Activation z-score	# Genes in dataset
Cancer,Gastrointestinal Disease,Organismal Injury and Abnormalities	Large intestine neoplasm	2.06E-11	Decreased	-2.19	712
Cellular Movement,Hematological System Development and Function,Immune Cell Trafficking	Cell movement of mononuclear leukocytes	0.000108	Decreased	-2.008	53
Organismal Survival	Organismal death	0.000202	Decreased	-2.348	216
DNA Replication, Recombination, and Repair	DNA replication	2.06E-06	Increased	2.077	35
Cell Death and Survival	Cell survival	9.69E-06	Increased	2.259	147
Cell Morphology,Cellular Function and Maintenance	Homologous recombination repair of tumor cell lines	1.46E-05	Increased	2.592	8
Cellular Growth and Proliferation	Colony formation of tumor cell lines	6.25E-05	Increased	2.109	39
DNA Replication, Recombination, and Repair	Metabolism of DNA	9.18E-05	Increased	2.011	44
Cellular Development,Cellular Growth and Proliferation,Hematological System Development and Function,Hematopoiesis	Proliferation of hematopoietic progenitor cells	0.000457	Increased	2.149	30
Cancer	Cell transformation	0.000851	Increased	2.442	45
Cellular Development	Epithelial-mesenchymal transition	0.00113	Increased	2.197	27
Hematological System Development and Function,Hematopoiesis,Tissue Morphology	Quantity of hematopoietic progenitor cells	0.00366	Increased	2.419	44

#### Hierarchical clustering of RNA sequence analysis

**Fig 4:**
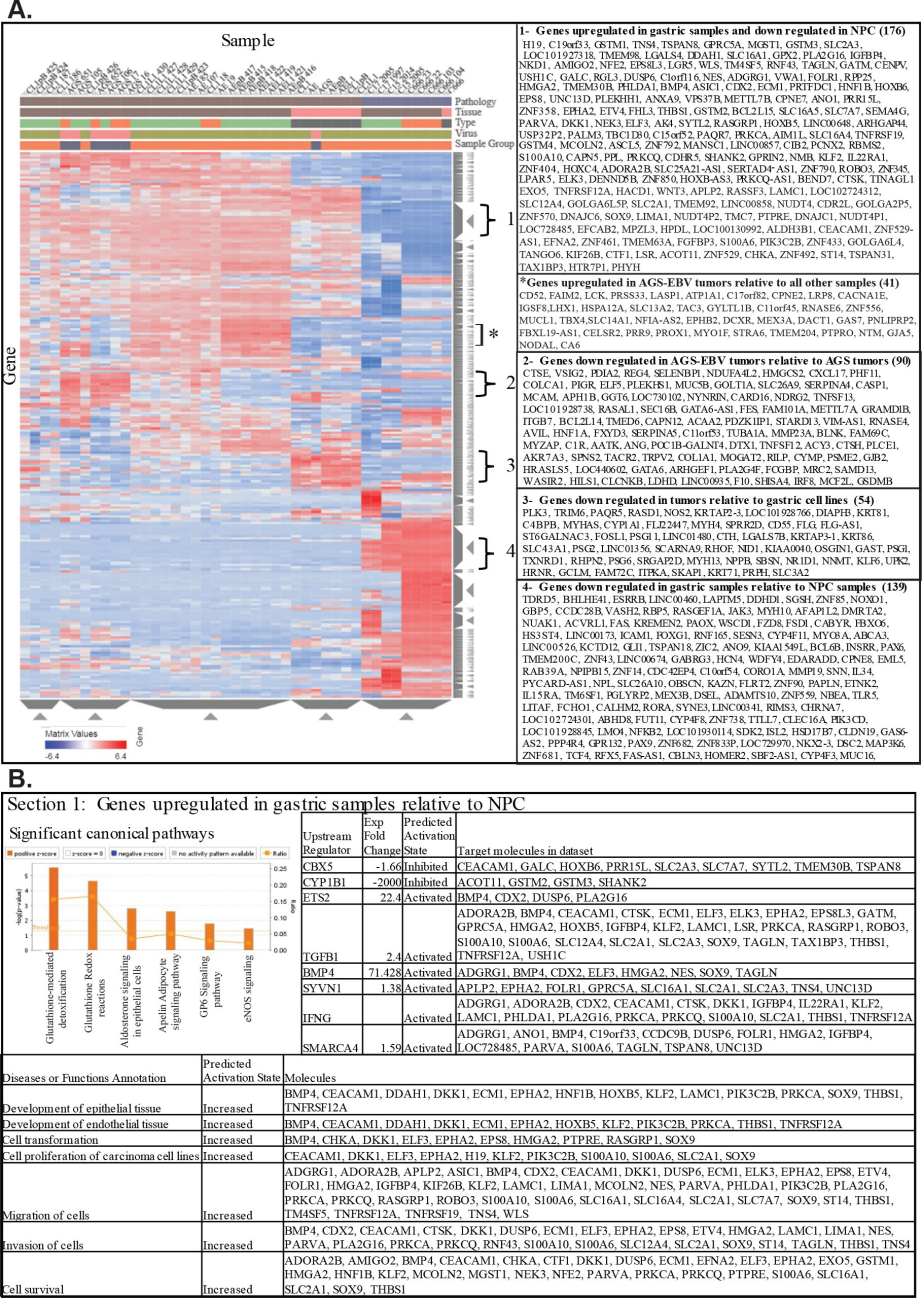
Hierarchical clustering heat map of gene expression of all samples and analysis of genes in distinct areas. (A) Hierarchical clustering heat map of gene expression vs hg19 and the associated genes. Red denotes upregulation and blue denotes downregulation. The samples are identified by pathology (gastric and NPC), Tissue (gastric tumor, cell line, NPC tumor), Type (sc tumor, ip tumor, cell line), virus (EBV negative and EBV positive), and sample grouping (EBV negative vs EBV positive). Genes from the five distinct areas are listed. (B) IPA analysis for canonical pathways, upstream regulators, and disease and functions of the genes from Section 1. Genes that are upregulated in the gastric samples relative to the NPC.

The RNA sequence data analyzed using hierarchical clustering based on differential expression of human genes clearly delineated the NPC from the gastric samples, the cell lines from the tumors, as well as the EBV negative gastric tumors from the EBV positive tumors (Fig 4A). Interestingly, the AGS cell line was completely within the cell line cluster containing the EBV positive cell lines as had also been indicated by the PCA analysis. Additionally, the C666 cell line clustered with the NPC tumors and was completely separated from the AGS cell lines. Several distinct expression patterns were visible on the hierarchical heat map (Fig 4A). Of the NPC samples, the C17 tumor was completely separated and had clear differences in expression. IPA analysis of one area (1) in which 176 genes were markedly increased in the gastric samples compared to the NPC samples, largely greater than four-fold, revealed that this region contained genes associated with cell proliferation, migration, invasion, and epithelial differentiation (Fig 4A-1). When the fold expression of these genes in this area as compared to NPC were analyzed by IPA, activation of canonical pathways involved in lipid metabolism (glutathione detox and redox), differentiation (aldersterone signaling, angiogenesis, adipocyte signaling and ERK/MAPK signaling), platelet activation (GP6 signaling), and cardiovascular signaling (Enos signaling) were predicted (Fig 4B). These pathways would lead to increased proliferation, transformation, migration, and invasion.

An area was also identified that included 41 genes that were upregulated specifically in the AGS-EBV gastric tumors relative to all the other samples. These genes included 8 which are involved in vasculogenesis; EPHB2, GJA5, LRP8, NODAL, PROX1, STRA6, TBX4, and TMEM204 (Fig 4A-*).

**Fig 5:**
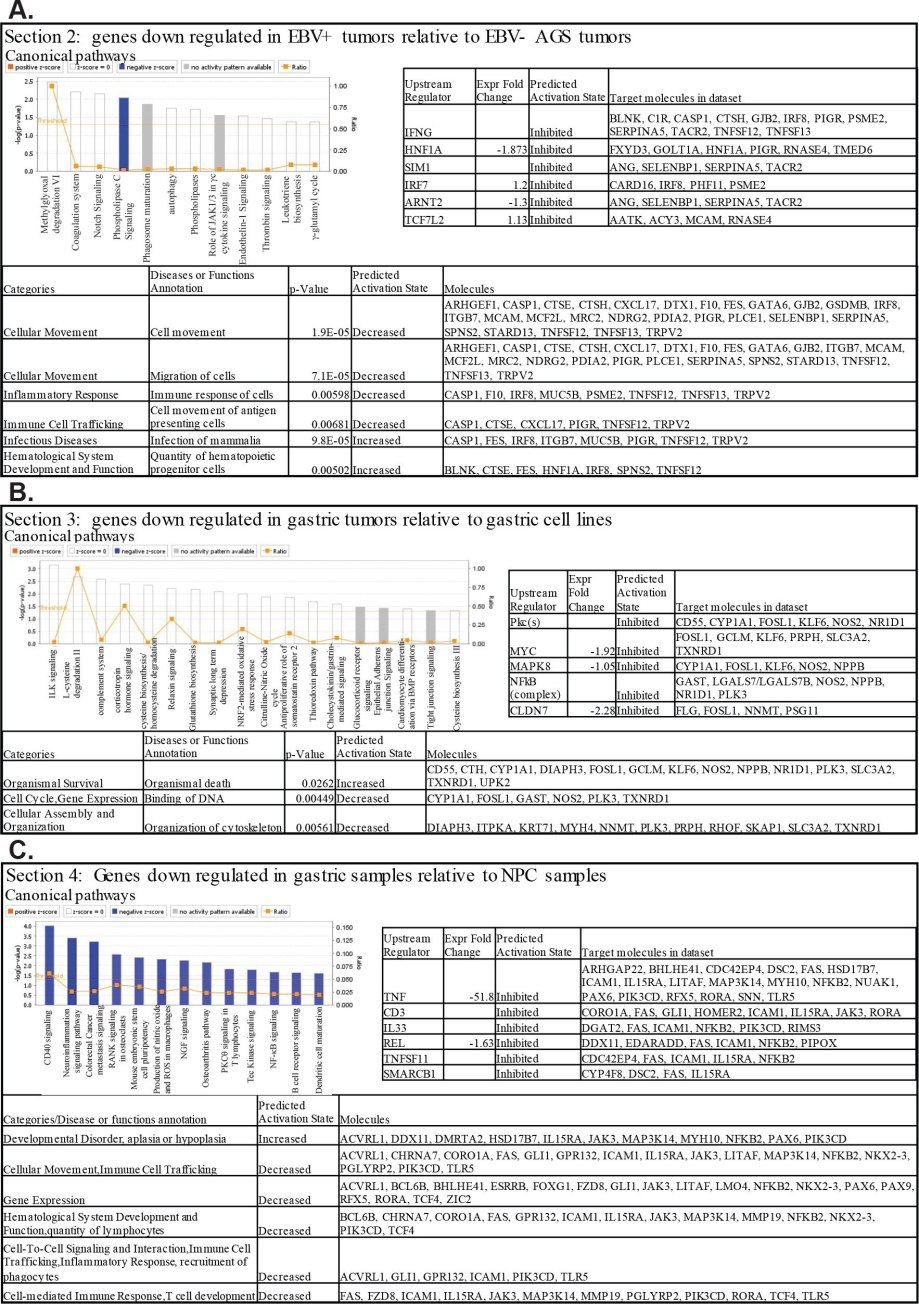
Analysis of canonical pathways, upstream regulators, and disease and functions within the annotated sections of the hierarchical clustering heat map. IPA analysis of the genes from 3 of the distinct areas on the hierarchical clustering heat map. (A) Section 2. Genes that are downregulated in the EBV positive tumors relative to the EBV negative tumors. (B) Section 3. Genes that are downregulated in the gastric tumors relative to the gastric cell lines. (C) Section 4. Genes that are downregulated in the gastric samples relative to the NPC samples.

The heat map also revealed an area with 90 genes which were down regulated in the EBV positive gastric and NPC tumors when compared to the EBV negative gastric tumors, and contained genes including CTSE (cathepsin E), CXCL17, ITB7 (integrin β7), PIGR, TNFS12, MUC5B (mucin), and TRPV2 that are involved in inflammatory response and movement of antigen presenting cells (Fig 4A-2). IPA analysis of the expression change of these 90 genes linked them to canonical pathways involved in lipid metabolism (methylgloxal degradation, phospholipase C signaling and phospholipases) as well as autophagy pathways (phagasome maturation and autophagy), hematological system development (Jak1 and JAK3 in cytokine signaling) and immune response (leukotriene biosynthesis). These pathways suggest that in the AGS-EBV tumors cell migration and inflammatory responses are decreased, and the quantity of hematopoietic progenitor cells are increased (Fig 5A). As described earlier the AGS-EBV tumors were noticeably bloodier than the AGS tumors upon gross examination (Table 1, Fig 1E).

A fourth area (Fig 4A-3) which had much higher expression in the cell lines contained 54 genes linked to amino acid metabolism (CTH, GCLM, and NOS2), migration (CYP1A1, FOSL1, GAST (gastrin), ITPKA, KLF6, NNMT,PSG1, SKAP1, and SLC3A2), and apoptosis and necrosis (CD55, CTH, FOSL1, GCLM, KLF6, NPPB, NR1D1, OSGIN1, PLK3, PRPH, PSG2, RASD1, and TXNRD1) (Fig 4A-3). The canonical pathways associated with these 54 genes involved metabolic pathways (L-cysteine, glutathione, thioredoxin, γ-glutamyl cycle, and citrulline-nitric oxide cycle) and integrin related signaling pathways (ILK, epithelial adherens junction, and tight junction signaling) and suggested within the tumors there is decreased cell survival, gene expression, and organization of the cytoskeleton as compared to the cell lines (Fig 5B).

A fifth area on the hierarchical heatmap contained 139 genes which were highly expressed in the NPC samples and decreased in the gastric samples. This region was composed of genes involved in CD40 signaling (MAP3K14 (NIK), ICAM1, PIK3CD, NFKB2 (p52), and JAK3), regulation of epithelial-mesenchymal transition (FZD8, TCF4, PIK3CD, NFKB2, and JAK3), and NFκB and PI3K signaling (MAP3K14, TLR5, PIK3CD, JAK3, and NFKB2), pathways shown to be activated in NPC (Fig 4A-4). The genes CHRNA7, CORO1A, FAS, ICAM1, IL15RA, JAK3, LAPTM5, MMP19, PGLYRP2, PIK3CD, and VSIR linked to activation of T lymphocytes were also more highly expressed in the NPCs, as well as the genes ACVRL1, AFAP1L2, CHRNA7, ETNK2, FAS, FZD8, IL34, INSRR, JAK3, MAP3K14, MAP3K6, MYO3A, NUAK1, OBSCN, PAX6, and PIK3CD involved in protein phosphorylation pathways.

IPA predicted the canonical pathways CD40 signaling, NFκB signaling, and B cell receptor signaling to be inhibited in the gastric samples relative to the NPC (Fig 5C). Immune cell trafficking and inflammatory response as well as gene expression were also predicted to be decreased in the gastric samples as compared to the NPC samples (Fig 5C).

The upstream regulators of the genes from these areas were also noticeably different. The genes that were significantly downregulated in the NPC samples (Fig 4A-1) as compared to the gastric samples that are involved in migration, invasion, and epithelial differentiation had predicted upstream regulators CBX5, CYP1B1, ETS2, TGFB1, BMP4, SYVN1, IFNG, and SMARCA4. Some of the regulators had major increases in expression in the gastric samples including ETS2, which was increased 22 fold in expression, BMP4, which was increased 71 fold, and IFNG (Fig 4B).

IRF7, SIM1, IFNG, and HNF1A were predicted to be upstream regulators of the higher expressed genes in the EBV negative gastric tumors involved in cellular movement, inflammatory response, and immune cell trafficking (Fig 5A). The genes that were significantly upregulated in the gastric cell lines as compared to the other samples and involved in amino acid metabolism, migration, apoptosis, adhesion, and necrosis had predicted upstream regulators CLDN7, HRAS, RNA pol 2 and PKcs (Fig 4A-3). When analyzed with the expression changes, the upstream regulators associated with these genes and that were predicted to be inhibited in the gastric tumors included the transcription regulators MYC and NFκB, the Pkc kinases and MAPK8 (JNK) as well as CLDN7 (Fig 5B).

ID2, ID3, TRAF3, TRAF5, REL, TNF and CD40, genes or pathways previously shown to be affected in NPC were the predicted upstream regulators for the pathways identified by the genes significantly upregulated in NPC on the heat map (Fig 4A-4) and involved in CD40 signaling, EMT, NFKB signaling, and PI3K signaling. When the 139 genes were analyzed with the fold expression changes, upstream regulators TNF and REL were predicted to be inhibited in the gastric samples as compared to the NPC (Fig 5C).

#### Genes consistently changed with EBV infection

There were 240 genes that were consistently changed in the same direction in all the EBV positive samples as compared to the EBV negative samples. These included 166 genes which were upregulated and 74 which were down regulated (S2 Table). The upregulated genes were predicted to lead to increased DNA repair and included the helicases DNA2, SMARCB1 and SMARCC; DNA repair genes MSH6, MCM6, and XRCC5; increased RNA transactivation and included transcription regulators GTF2H3, CCNT1, RARA, KHDRBS1, VOPP1 and NFE2L1; increased viral infection, and decreased survival (S3A Table). Several proto-oncogenes were upregulated in the EBV positive samples, including MYBL1 and RAF1 (S2 Table). Several kinases were also upregulated including NCK1, PRKACA, and TK1 (S2 Table). The consistently downregulated genes were predicted to lead to decreased movement, survival, proliferation, and immune response and increased apoptosis (S3B Table). The down regulated genes included two serine protease inhibitors SLPI and SERPINA3, cell adhesion genes CEACAM5 and CEACAM6, transcription regulators ELF3 and GATA6 (S2 Table).

#### Comparison of the top 100 downregulated and upregulated genes in the tumors and cell lines

**Fig 6:**
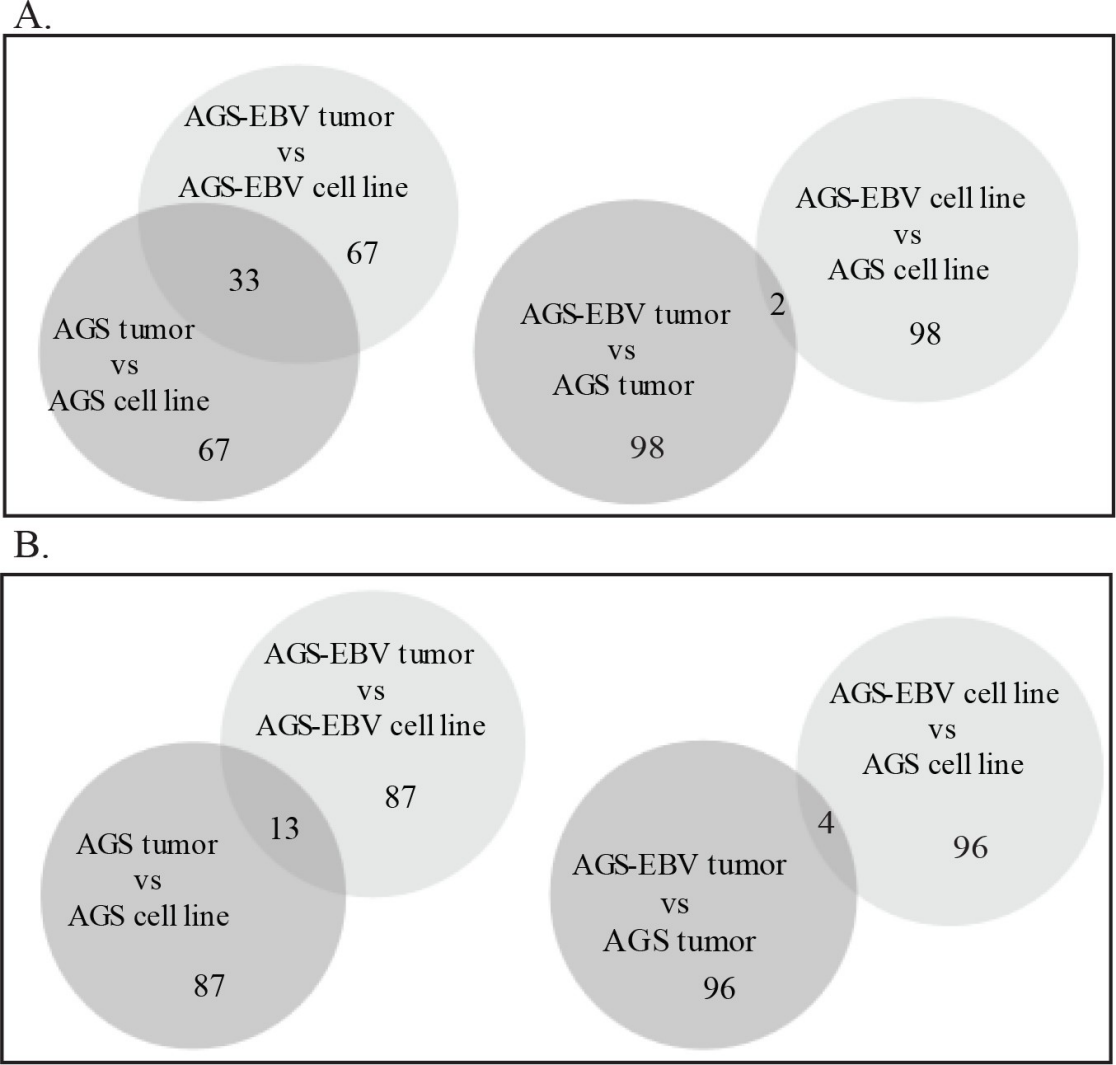
Comparison of the top 100 down regulated and upregulated genes in the data sets. A. Venn diagrams showing comparisons of the top 100 down regulated genes in each data set presented in S4 Table. B. Venn diagrams showing the comparison of the top 100 up regulated genes in each data set presented in S4 Table.

To provide a more detailed look at the specific genes composing each data set, the top 100 down regulated and upregulated genes in each comparison are listed (S4 Table). These data were then utilized to prepare Venn diagrams revealing the extent of overlap between each group (Fig 6). The comparisons reveal that of the 100 genes downregulated in the AGS tumors compared with the AGS cell line, 33 are in common with the 100 genes downregulated in the AGS-EBV tumors compared with the AGS-EBV cell line (Fig 6A). Analysis of the upregulated genes revealed that 13 were in common between the AGS-EBV tumors and AGS tumors (Fig 6B). Of the 100 genes decreased in the AGS-EBV tumors compared with the AGS tumors, only 2 are in common with the 100 genes decreased in the AGS-EBV cell lines compared to the AGS cell line (Fig 6A) while in the 100 upregulated genes 4 are in common between those affected in the AGS-EBV tumor and the AGS-EBV cell line (Fig 6B). When the top 100 up regulated genes in the AGS-EBV cell lines as compared to the AGS cell line were analyzed, many are involved in viability and organization of the cytoplasm (S5A Table). Morbidity and Mortality is predicted to be strongly decreased. It is likely that many of these functions are linked to the upregulation of BCL6 and TCF3 in the AGS-EBV cell line. The upregulated genes in the AGS-EBV tumors are predicted to increase migration and movement as well as amino acid phosphorylation (S5B Table). These classifications may reflect the upregulation of FYN and LCK which are molecules identified in every category.

#### EBV transcription with identified splice junctions

**Fig 7:**
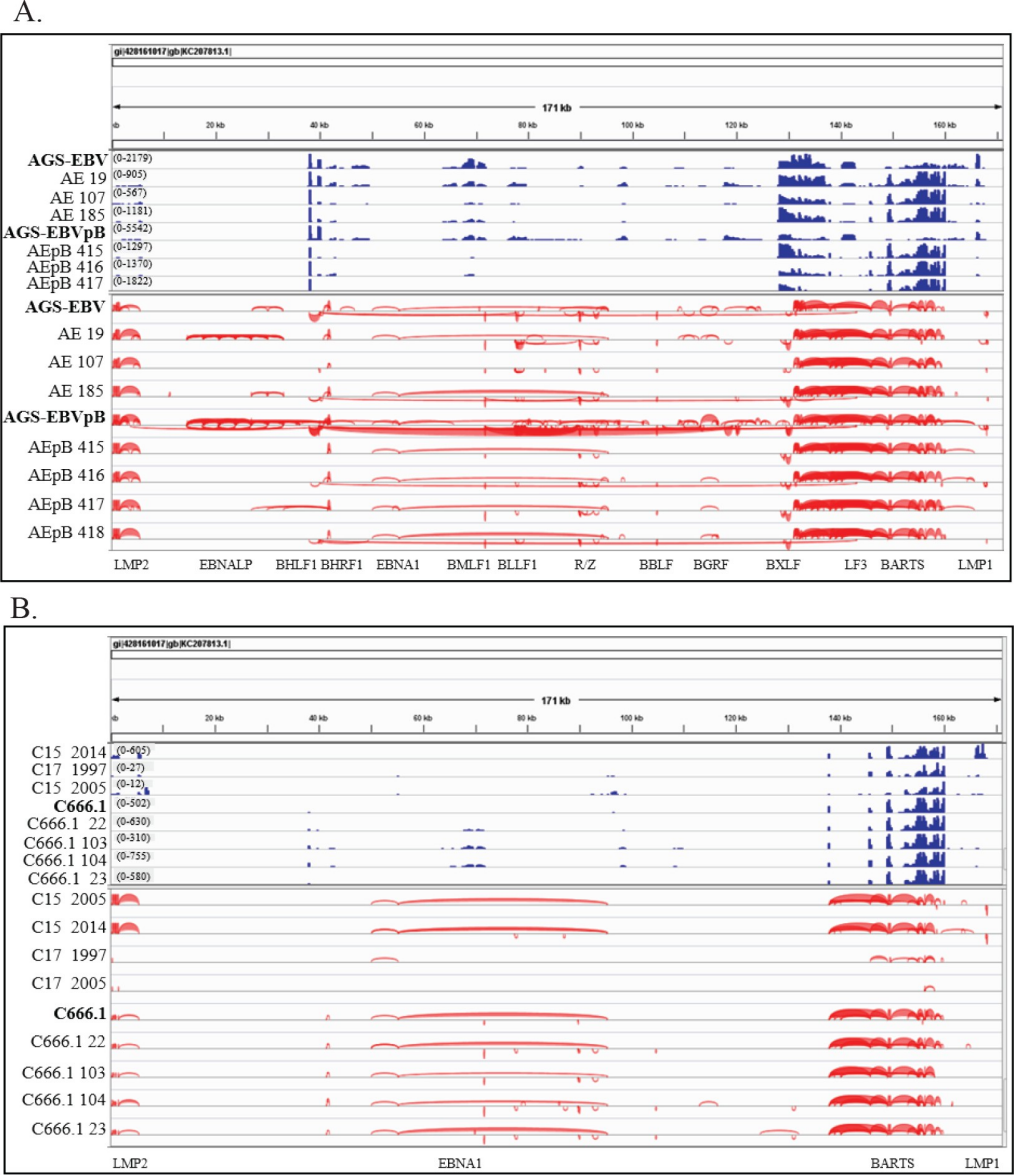
Visualization of the EBV reads and splice junctions. (A) Mapped reads and splice junctions of AGS-EBV cell lines and representative tumors mapped to the Akata genome. The scale in parentheses represents the number of reads with the height of the blue peaks also representing the abundance of reads. The EBV splice junctions are shown below in red with their corresponding EBV gene denoted on the bottom axis with the thickness of the red lines indicating the frequency of detection of specific splices. (B) Mapped reads and splice junctions of NPC cell line and tumors mapped to the Akata genome. The scale in parentheses represents the number of reads with the height of the blue peaks also representing the abundance of reads. The EBV splice junctions are shown below in red with their corresponding EBV gene denoted on the bottom axis with the thickness of the red lines indicating the frequency of detection of specific splices.

To determine the EBV specific transcription, reads that did not map to the human and mouse genomes, were aligned to the Akata EBV sequence using Tophat. The transcripts were assembled using Cufflinks and further assembled and quantified using Cufflinks and Stringtie (Fig 7). Comparison of the AGS-EBV cell line and tumors did not reveal any obvious differences with transcription of the same sequences. The most abundantly transcribed region was from the BART noncoding locus (Fig 7A and Table 7). Identification of the specific transcripts revealed that the AGS-EBV cell line was relatively permissive with expression of replicative genes from both strands. Additionally, multiple transcripts were identified that spliced into the BART locus. Comparison with the NPC samples, C15 and C666, revealed that the NPC samples remained tightly latent with considerably lower levels of transcription (Fig 7B). The C15 tumor had a scale of 605 EBV reads while the AGS-EBV sample had a scale of more than 2000 reads. In contrast to the AGS-EBV cell line, the tumors had more restricted expression with decreases in number of detected reads (Table 2 and Fig 7A). The tumors had consistent expression of the BART RNAs, EBNA1 and LMP2. Additionally, the cell lines had greater evidence of transcription of replicative genes from the opposite strand while the tumors retained some replicative expression. Interestingly most of the tumors had consistent expression of BHRF1 and BHLF1 and some transcription of R/Z. The C15 and C666 tumors were similar with consistent expression of the BART RNAs, EBNA1, and LMP2 while reads from BHRF and BHLF1 were detected in C666 tumors and LMP1 in C15 (Fig 7B and Table 7). In almost all of the gastric tumors, there was an increase in reads from the BARTs when compared to the cell lines, and fewer reads from BHLF1, EBNA1 and R/Z, and LMP2 (Fig 7A and S4 Fig). Interestingly abundant transcription was detected immediately upstream of the BART region at approximately 130 kb on the EBV genome only in the AGS-EBV cell lines and tumors (Fig 7A and S4 Fig). In the tumors from 13–100% of the previously described BART transcripts contained exons splicing from this upstream area. This region of transcription contains multiple putative ORFs. The AGS-EBV and Clone 1 cell lines and tumors also had a considerable peak of reads between 140 and 143 kb on the Akata genome within the LF3 region (Fig 7A and S4 Fig). Analysis of splice junctions indicated that in cell lines with abundant replicative expression, transcripts could be detected that spliced from EBNA1 into the BART complex (Table 7). Additional aberrant splice junctions were detected including BXLF/BDLF to BHLF, BNLF/LF3 to BHLF1, and oriLyt/LF3 to BHLF. These splices may indicate decreased regulation of splicing during replication or potential detection of circular RNAs that have been detected by RNA Seq [16]. However, the abundant and consistent expression of the BART transcripts suggests that they are likely major effectors of EBV on cellular expression in both the EBV infected AGS cell line and AGS and NPC tumors.

**Table 7:** Average percent of EBV spliced transcripts from Cufflinks and Stringtie*.

Sample	LMP2	EBNALP/ EBNA2	EBNA3	BHLF1	BHRF1	EBNA1	BSLF/ BMLF	Z/R-Z	BBLF	BGRF/ BDRF	BXLF gH/TK	BARTs total	% BARTs with upstream exons	LF3	LMP1
**AGS-EBV**	4.9			3.7	10.9	13.4		8.0	6.7			44.7	100.0		7.8
AE 19	6.4			1.1	29.3	5.4	3.3	4.8	4.3	1.1		39.8	50.0		4.6
AE 107	17.5			1.7	12.1		2.2	2.2	0.7			61.4	40.8		2.3
AE 185	11.1			0.7	16.2	2.3	7.4	2.0	0.9			59.5	37.9		
**AEpB**	23.3		13.3	4.6^1^		4.9		13.2			4.6^1^	25.5	72.6		14.9
AEpB 415	10.0			2.1	5.0	1.1	0.9	0.6			0.6	79.7	94.0		
AEpB 416	13.4			12.6	18.5	0.5	1.1	2.1	1.1	0.4		50.3	47.8		
AEpB 417	16.6			3.3	28.4	0.9	3.7	1.4	0.9	0.5	0.6	43.7	100.0		
AEpB 418	7.9			1.7	17.4	1.6	2.4	1.6	1.2	0.9	0.5	64.7	50.0		
**AEL1**	6.3			0.3	18.5	5.7	15.1	5.1	9.2	4.7		23.3	74.0		11.8
AEL1 419	5.3			4.9	19.9	2.8	4.3	1.9	1.3	0.3		59.2	44.3		
AEL1 421	8.4			1.1^2^	12.8	1.0	0.5	0.9	0.3	0.3	0.3	74.3	63.3	0.5^2^	
AEL1 422	16.8			4.8		9.8	2.4	4.8	0.7		0.8	60.0	23.6		
**Clone1**	48.6	5.8			27.5^3^			3.8	0.5		1.2	11.3^3^	100.0		1.5
CL1 18	6.8			7.0	3.1	0.8	2.1	1.1			1.4	77.8	60.4		
CL1 186	9.1			8.1	33.9	5.0	15.9	2.2	6.3			19.5	92.1		
CL1 187	10.0			0.2		26.5		12.8	16.9	6.3		18.8	89.9	0.5	8.1
**CL1pB**	14.9	10.0	0.8	16.3^4^	12.5^5^	7.4		9.7				18.9	66.3^5^	12.1^4^	9.3
CL1pB 423	10.2			2.3	23.7	3.7	7.0	3.7	4.8	0.8		43.7	54.8		
CL1pB 424	3.4		0.1	10.3^6^	45.9	1.2	6.3	2.3	5.0	0.9		24.6	13.7	10.3^6^	
CL1pB 425	15.4			1.7	24.4	8.5		5.5	12.2		0.2	32.1	50.0		
CL1pB 426	24.9			4.0	19.5	1.9	14.2	4.6	1.6	9.5		19.6	50.0		
**CL1L1**	8.9			1.2	0.3	19.3	3.1	9.8	4.4	11.8		35.6	78.9		5.5
C1L1 427	11.5			1.6	42.3	4.9	7.0	3.1	6.3	5.0		12.6	47.0		5.8
C1L1 428	9.9			2.7	32.0	3.9	6.7	4.6	13.0			27.1	34.9		
C1L1 429	5.5			3.7	30.8	12.3		3.8	3.7			40.2	36.4		
C1L1 430	5.7			0.3	36.4	6.3			2.1	5.1		44.1	92.0		
**C666.1**	3.3			3.4	0.4	2.2	0.7					90.0			
C666 22	4.9			2.1	5.0	4.0	9.0	1.6	0.2			73.1			
C666 23	2.6			2.9	1.4	2.6	4.4	0.5	0.5		0.1	85.0			
C666 103	3.1			2.0	5.2	2.3	7.5	1.6				78.2			
C666 104	6.1			2.2	8.2	1.8	12.9	2.8	1.1	1.3	1.0	62.6			
C15 2005	17.0					2.6						33.1			47.4
C15 2014	12.4					3.8						74.8			9.0
C17 1997												100.0			
C17 2005												100.0			

*Based on FPKMs (Fragments Per Kilobase of transcript per Million mapped reads) Total FPKM of distinct transcript/total FPKMs of all transcripts (average of Cufflink and Stringtie)

^1^ BHLF splices from BXLF/BDLF in AEpB (130435–119179 v 40093–37879)

^2^ 50% BHLF1 splices from BNLF/LF3 in AEL1 421 (169339–143022 v 40093–37252)

^3^ 60% of BART transcripts splice upstream from BWRF/BHRF in Clone1

^4^ 75% of BHLF transcripts splice from orilyt/LF3 to BHLF in Clone1pB

^5^ BART transcripts splice upstream from BWRF/BHRF in Clone1pB

^6^ BHLF transcripts splice from orilyt/LF3 to BHLF in CL1pB 424 (146303–143022 v 40093–37879)

The percentage of specific EBV reads indicates that both the AGS-EBV cell lines and tumors had expression of genes associated with viral replication (Table 7). One distinction is the greatly increased number of reads in the tumors representing BHRF1, the EBV bcl2 homologue [17]. The other notable feature was the increase in reads from the BART region, which frequently represented 40–70% of transcription. In the C666 cell line, 90% of the reads were BART transcripts while in C17, only BART transcription was detected with 100% of the reads from the BART region. All of the AGS-EBV and Clone 1 tumors and cell lines had considerable expression of LMP2 (Table 7).

### Quantitative expression of EBV transcription during tumor growth

**Fig 8:**
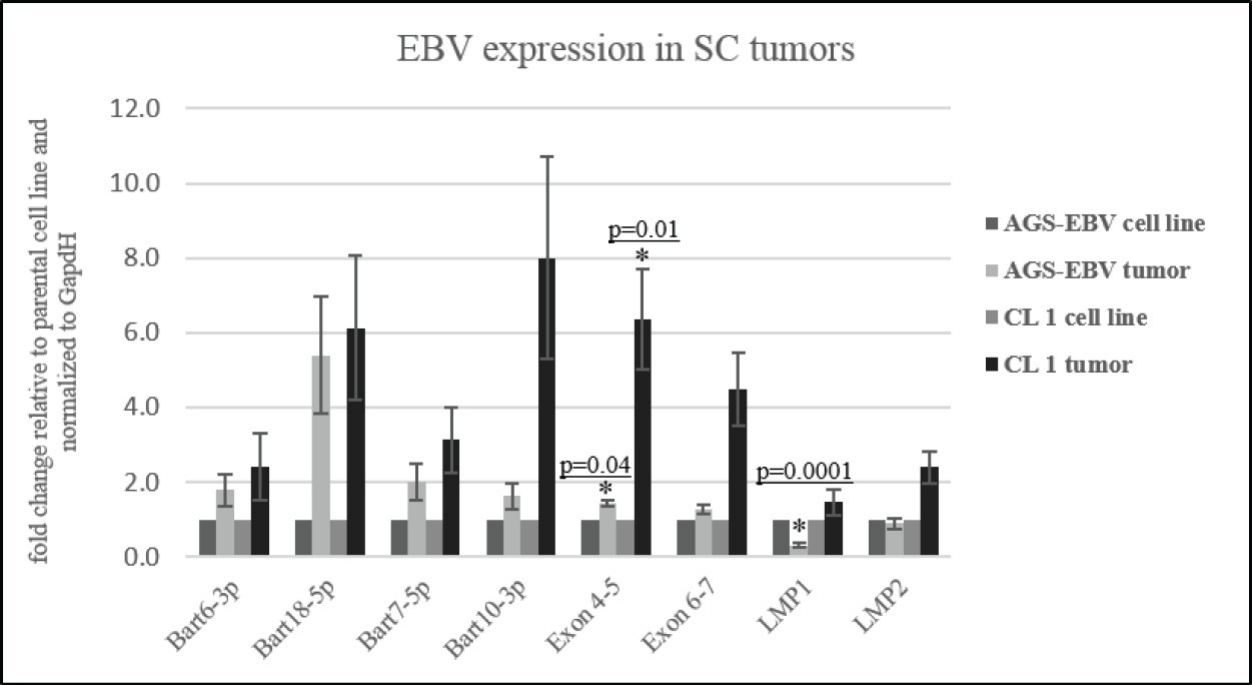
Quantitative PCR for representative BART miRs, splices for the BARTlnc transcripts, and EBV LMP1 and LMP2. Quantitative PCR comparing the fold change in expression in the AGS-EBV tumors in reference to the AGS-EBV cell line, and in the Clone 1 tumors in reference to the Clone 1 cell line, with the significant *p* values denoted.

A previous study had suggested that BART miRNA expression increased during tumor growth *in vivo* [13]. To evaluate levels of expression, quantitative PCR was performed for several BART miRNAs, for two splices characteristic of the BART lnc RNAs between exons 4 and 5 and 6 and 7, and for LMP1 and LMP2 (Fig 8). BART miRNAs increased approximately two fold to eight fold while the lnc splice junctions increased approximately 1.5 to 5 fold with significant p values. Expression of LMP1 and LMP2 remained low although they increased in clone 1 tumors which also had increased replicative expression.

## Discussion

In this study, AGS cells with or without EBV were inoculated into immunodeficient mice either subcutaneously or intraperitoneally. All inoculations formed tumors with slightly enhanced tumor growth in the cells containing EBV. Importantly, although the cells were not maintained under G418 selection, which is required to maintain the EBV genome *in vitro*, all EBV positive tumors contained and expressed viral genes without selection. This finding suggests that EBV is providing a survival advantage to the inoculated cells [18]. This is perhaps related to the finding that NPC tumors usually lose the EBV genome when cultured *in vitro* [6].

An intriguing difference in the EBV positive gastric tumors was the greatly increased amounts of blood and hemorrhage that was not greatly evidenced in the expression analyses. Identification of disease and function did identify increased hematologic system development and function (Table 6). A recent study identified vasculogenic mimicry in EBV-associated epithelial malignancies where the tumor cells develop vascular networks [19]. The increased vascularization identified in our study substantiates this and that this difference was not due to infiltrating mouse cells (Table 2). However, the previous study linked this vascularization to activation of the Akt and HIF1-α pathways that were not identified in the analyses here where HIF1-α was predicted to be inhibited (Table 5). Activation of Akt was identified in Biojupes analyses that looked at the top 500 genes with increased expression. However, the previous study that identified vasculogenic mimicry primarily focused on NPC where LMP1 and LMP2 that are known to activate these pathways are expressed [6, 20]. In that study, the analyses of gastric tumors did identify activation of Akt and HIF1-α using immunohistochemistry on tumor samples. However, the expression of other viral genes was not assessed [19]. It is possible that the same biologic effectors of Akt and HIF1-α are activated in the AGS-EBV gastric tumors through subtle effects of the noncoding RNAs.

Importantly, the data revealed major changes in cellular expression when grown as tumors in immunodeficient mice compared to cells cultured *in vitro*. Analysis of the global changes of expression greater than two fold revealed that many canonical pathways had significant numbers of genes with altered expression. Many of the genes might be expected as they would contribute to growth *in vitro*. In most of the identified pathways activation or inhibition was not apparent. Although the many pathways that are not predicted to be activated or repressed, the considerable number of genes within these pathways and the very significant p values suggest that they are altered during tumor growth. However, the majority of pathways that were significantly affected in both the EBV positive and negative AGS tumors were predicted to be repressed. The mice lack an immune system so that immune selection is unlikely, however, these findings suggest that there is an advantage to not have activation of the multiple pathways that are increased *in vitro*. Intriguingly, identification of the predicted upstream regulators within the tumors revealed induced expression of miRNAs. This suggests that noncoding RNAs are likely key regulators of the global changes in expression during tumor growth in the analysis of total transcriptional changes. It is possible that the noncoding RNAs may broadly affect cellular expression perhaps through epigenetic mechanisms [12]. Additionally, HDAC1 is revealed to be an EBV unique upstream regulator in the AGS-EBV tumors also confirming the shift to epigenetic regulation during tumor formation (Table 4).

In contrast, hierarchical clustering revealed clear distinction that delineated the specific groups, NPC tumors, cell lines, and EBV positive and negative tumors (Fig 4). The genes identified within the delineating regions identified activation of key biologic functions including differentiation, motility, metastasis, transformation. Importantly, most of the fold expression changes within the delineating regions were greater than 4 fold. In the gastric tumors, many of these genes are regulated by ETS2, BMP4, and TGFB1 and by the kinases MAPK1(ERK) and CCNK and the transcription regulators E2F, CBX5, and CCND1. The EBV negative tumors, the pathways were regulated by HNF1A, SIM1, IRF7, TCF7L2, and SMARCA4, as well as IFNG, CLDN7, and let-7.

The EBV positive samples also showed consistent up regulation or down regulation of certain genes, 240 in all, when compared to the EBV negative samples (S2 Table). Many of the upregulated genes are involved in DNA replication and repair, RNA transactivation, and cell cycle progression, all programs that suggest a more proliferative state in the EBV infected tumors (S3 Table). Interestingly, 2 proto-oncogenes, MYBL1 and RAF1 were consistently upregulated in the EBV positive tumors, and NACC1, which negatively regulates the tumor suppressor Gadd45/GIp1, was consistently upregulated (S2 Table). Several of these upregulated genes are targets of miR-122 and let-7, both predicted to be uniquely activated in the EBV negative samples. Many of the genes down regulated in the EBV positive tumors are involved in immune response and movement. Interestingly, several of the genes such as TRIM24, EGLN3, FUT1, MUC20, GATA6, and S100P that were consistently down regulated in the EBV positive samples are also targets of the BART miRs (S2 Table) [21]. Also other predicted BART miR targets including DICER, CASP3, CDH1, TOMM22, and PAK2 were consistently down regulated (less than 2 fold) in the EBV positive gastric cell lines and tumors as compared to the EBV negative [22]. This data suggests that the BART miRs may regulated gene expression not only at the translational level, but also at the transcriptional level. Another interesting comparison is identification of genes whose expression is changed in the same direction as within an EBV cell line expressing one form of the spliced BART lnc RNAs (S6 Table) [11]. Several of the downregulated genes, TFF1, SLPI, and MUC1 are found at the mucosal barrier; others are involved in cell adhesion, CEACAM5, MSLN, and PTPRH; and RNF144B is proapototic (S6 Table). Of the possible BARTlnc targets that were upregulated, several, MPP2, S1PR2, and TSPAN9 are involved in proliferation, growth and motility (S6 Table). In most cases, the genes affected in the lnc cell line were affected to a much greater level within the tumors. This suggests that within the tumors in the presence of all forms of the spliced BART RNAs and the BART miRNAs, the effects are considerably greater.

These data provide us new insight into EBV expression. The expression *in vivo* reveals how tightly latent the EBV NPC tumors are and that EBV leaky replicative expression remained detectable in the AGS-EBV tumors *in vivo*. The significance of the unusual splice linkages is unclear. They are not overwhelmingly abundant but may not be tabulated completely in this analysis. One intriguing fact is the more frequent linkage of orilyt/LF3 to BHLF1. An abundant circular RNA has been inferred from sequence analyses [16]. The linkage between LF3 and BHLF1 may represent an unusual RNA, perhaps circular, that functions during replication. It is possible that this RNA may contribute in some replicative way to lytic origin function. However, overall these data affirm the importance of latent infection in tumorigenesis and strongly underscore the significance of the BART nc RNAs. It is possible that the tumor cells are epigenetically poised for further activation of expression due to the viral nc RNAs and that these global epigenetic effects on cellular expression are a major mechanism through which EBV contributes to tumorigenesis.

## Materials and methods

### Ethics statement

All animals at the University of North Carolina are maintained in compliance with the Animal Welfare Act and the Department of Health and Human Service “Guide for the Care and Use of Laboratory Animals.” UNC’s Animal Welfare Assurance Number is A3410-01. Animal experiments were performed in accordance with a protocol (#17–031) approved by the Institutional Animal Care and Use Committee (IACUC) at the University of North Carolina. Mice were monitored daily following injection for signs of distress and tumor growth. At all monitoring intervals, post-injection general appearance and body weights were recorded. When the tumors reached approved endpoint growth or the animals were in distress the animals were euthanized by exposure to CO2 in a chamber, followed by injection of dormitory/ketaset (300mg/kg and 3mg/kg weight).

#### Cell lines

AGS (EBV negative gastric carcinoma) cell line was grown in F-12 media (Life Technologies, Inc.) with 10% fetal bovine serum and antibiotic/antimycotic (Life technologies, Inc.), AGS-EBV (EBV+ gastric carcinoma (infected with EBV Akata BX1) and Clone 1 (EBV^+^ clone from AGS-EBV cell line have been previously described [23] and were maintained with 500ug/ml G418 (Life Technologies, Inc.). Stable AGS-EBV and Clone 1 cell lines containing EBV LMP1 were generated by transduction of the pBabe control or HA tagged pBabe-LMP1(B95 strain) constructs and selected with 1ug/ml puromycin; and the C666.1 (EBV^+^ NPC) cell line was maintained in RPMI 1640 (Life Technologies, Inc.) with 10% fetal bovine serum and antibiotic/antimycotic (Life Technologies, Inc.) [24].

#### Injections

1 x 10^7^ cells were injected either intraperitoneal (ip) or subcutaneous (sc) into NOD scid gamma (NSG) mice. Mice were monitored for tumor growth and illness, and tumor and spleen tissues were harvested at the end point. Splenocytes from a primary tumor that developed from the injection of the C666.1 cell line were purified and reinjected into 2 NSG mice.

#### RNA sequencing

RNA was prepared from tumors that developed in the mice using Trizol reagent (Life Technologies, Inc). Poly(A)-selected, bar-coded, cDNA libraries were prepared using a TruSeq Stranded mRNA kit (Illumina) and the libraries were sequenced using a HiSeq4000 instrument (Illumina) using paired-end 75-bp sequencing by the UNC High-Throughput Sequencing facility. RNA was also prepared and analyzed as above from tumors from the NPC xenografts C15 and C17.

#### Bioinformatics

RNA sequencing reads were aligned to the human genome (hg19) and the mouse genome (mm9) using the splicing aware read aligner TopHat on the Galaxy suite at https://usegalaxy.org [25]. Aligned reads were mapped to specific human or mouse Refseq genes using the Partek Genomics Suite, which was also used to calculate differentially expressed genes and to perform principal component analysis. In order to quantify and visualize reads from the EBV Akata genome, RNA sequencing reads that did not map to the human genome were aligned to the Akata genome (Genbank accession number KC207813.1) using TopHat, and transcripts assembled using Cufflinks, and further assembled and quantified using Stringtie on the Galaxy suite. EBV aligned transcripts were visualized from the TopHat assemblies using the Integrative Genomics Viewer. Enriched molecular functions and pathways for the human and mouse data were obtained by running a core analysis on the differentially expressed genes using Ingenuity Pathway Analysis (IPA) software (Qiagen).

The gene rpkms against the human and mouse genomes were also analyzed in Biojupies, a web based program available at https://amp.pharm.mssm.edu/biojupies/ [26].

Hierarchical clustering maps and volcano plots were generated. Upstream regulators (transcription factors and kinases) as well as cellular pathways were determined and used to strengthen the data from the Partek and IPA analysis.

The RNA sequencing files for the transcriptome analysis of the gastric samples are available at SRA accession PRJNA503182. The RNA sequencing files for the transcriptome analysis of the NPC samples are available at SRA accession PRJNA501807.

#### Western blotting

Protein lysates were prepared from the tumor tissue, by re-suspending pulverized tissue in RIPA buffer. Western blotting was performed as previously described [27]. Primary antibodies used were mouse α-PCNA (Santa Cruz, sc-56) and mouse α-HSC70 (Santa Cruz, sc-7298).

#### Quantitative RT-PCR

Total RNA was prepared from the tumors and cell lines using Trizol reagent (Invitrogen). Quantitative RT-PCR was performed for the BART miRNAs and the BART lncRNA using the miScript system (Qiagen) and Quantifast SYBR green RT-PCR kit (Qiagen) on a QuantStudio 6 Flex real-time PCR system (Applied Biosystems) as described previously [28].

#### 2-D Difference gel electrophoresis

Proteins isolated from cell or tumor samples were cleaned by methanol/chloroform precipitation and dissolved in lysis buffer (9M urea, 2M Thiourea, 20 mM tris-HCl, pH 8.5, 2% CHAPS, and 60 mM n-Octyl-β-D-glucopyranoside). Aliquots (35 μg) of replicates from each tumor or cell line group were labeled with 200 pmol either Cy3 or Cy5 fluorescent dyes, alternating Cy3 and Cy5 between replicates. An internal control (IC) was prepared by pooling equal amounts of protein (18.5 μg) from all samples, and then labeled with 200 pmol of Cy2 for every 15 μg of protein. The labeling reaction was carried out on ice for 30 min, protected from light, and quenched by the addition of 1 μL of 10mM followed by 10 min on ice. After labeling, corresponding samples were combined so that each gel contained an IC (Cy2) and two samples from different groups (Cy3 and Cy5). An equal volume of 2X sample buffer (9M urea, 2M Thiourea, 2% CHAPS, and 60 mM n-Octyl-β-D-glucopyranoside, 30 mg/mL DTT, 2% (v/v) IPG buffer 3–10) was added and the mixture was placed on ice for 15 min. Rehydration buffer (9M urea, 2M Thiourea, 2% CHAPS, and 60 mM n-Octyl-β-D-glucopyranoside, 15 mg/mL DTT, 2% (v/v) IPG buffer 3–10) was then added to a final volume of 450 μL.

The resulting mix was loaded onto an immobilized pH gradient (IPG) strip (24 cm, pI range 3–10) and the strip allowed to re-hydrate overnight at room temperature. Isoelectric focusing and the subsequent SDS-PAGE, using precast 12.5% gels (Jule Biotechnologies Inc.), were performed as originally described [29]. Following SDS-PAGE gels were scanned using a Typhoon Trio Plus scanner (GE Healthcare) and analyzed using DeCyder 7.0 software (GE Healthcare).

## Supporting information

S1 Fig Canonical pathways predicted for the AGS-EBV tumors vs AGS tumors. Canonical pathways associated with human genes with 2-fold expression change by RNA-seq in the AGS-EBV and Clone 1 and pB derivative tumors when compared to the AGS tumors. (A) Top canonical pathways associated with AGS-EBV tumors compared to AGS tumors. (B) Significant canonical pathways (absolute z-score ≥2) associated with AGS-EBV tumors compared to AGS tumors. The height of the bars reflects the p value, and the orange boxes reflect the ratio of the number of genes in the data set represented in the pathway.
(TIF)

S2 Fig Canonical pathways predicted for C666.1 tumors vs C666.1 cell line. Top canonical pathways associated with human genes with 2-fold expression change by RNA-seq in the C666.1 tumors when compared to the C666.1 cell line. The height of the bars reflects the p value, and the orange boxes reflect the ratio of the number of genes in the data set that are represented in the pathway.
(TIF)

S3 Fig Significant canonical pathways predicted for the NPC tumors vs gastric tumors. A. Significant canonical pathways predicted for the NPC tumors vs the AGS tumors. Significant canonical pathways (absolute z-score ≥2) associated with the human genes with 2-fold expression change by RNA-seq in the NPC tumors when compared to the AGS tumors. The height of the bars reflects the p value, and the orange boxes reflect the ratio of the number of genes in the data set that are represented in the pathway. Astericks (*) denote pathways unique to the comparison of NPC tumors to AGS tumors. B. Significant canonical pathways predicted for the NPC tumors vs the AGS-EBV tumors. Significant canonical pathways (absolute z-score ≥2) associated with the human genes with 2-fold expression change by RNA-seq in the NPC tumors when compared to the AGS-EBV tumors. The height of the bars reflects the p value, and the orange boxes reflect the ratio of the number of genes in the data set that are represented in the pathway. Astericks (*) denote pathways unique to the comparison of NPC tumors to AGS-EBV tumors.
(TIF)

S4 Fig Visualization of the EBV reads from the EBV^+^ gastric tumors. Mapped reads of AGS-EBV cell lines and tumors mapped to the Akata genome. The number of reads correlate with the height of the blue peaks.
(TIF)

S1 Table Predicted common and unique upstream regulators in gastric tumors vs cell lines. (DOCX)

S2 Table Genes changed in the same direction in all EBV^+^ samples as compared to EBV^-^ samples. Shown are the genes consistently upregulated or down regulated in all the EBV^+^ samples with the fold expression change.
(DOCX)

S3 Table Disease and functions predicted for the 240 genes consistently changed in EBV^+^ samples. A. Disease and functions predicted by IPA for the 166 genes upregulated in all EBV^+^ samples. B. Disease and functions predicted by IP for the 74 genes down regulated in all EBV^+.^
(DOCX)

S4 Table Top 200 changed genes in each data set. A. List of top 100 down regulated genes in each data set and the fold change range. B. List of the top 100 upregulated genes in each data set and the fold change range.
(DOCX)

S5 Table Disease and functions of the top 100 upregulated genes in the AGS-EBV cell lines and tumors. A. Disease and functions of the top 100 upregulated genes in the AGS-EBV cell lines compared the AGS cell line. B. Disease and functions of the top 100 upregulated genes in the AGS-EBV tumors compared to the AGS tumors.
(DOCX)

S6 Table Correlation with potential BARTlnc targets. List of genes changed at least 1.5 fold in the EBV^+^ cell lines, EBV^+^ tumors and the BART cell line [11] when compared to the EBV^-^ control. P values and fold changes are denoted.
(DOCX)
