# Supplementary figures and images for: Alterations in cellular expression in EBV infected epithelial cell lines and tumors
Source: PLoS Pathog. 2019 Oct 4;15(10):e1008071. doi: 10.1371/journal.ppat.1008071 (PMC6795468; doi:10.1371/journal.ppat.1008071)

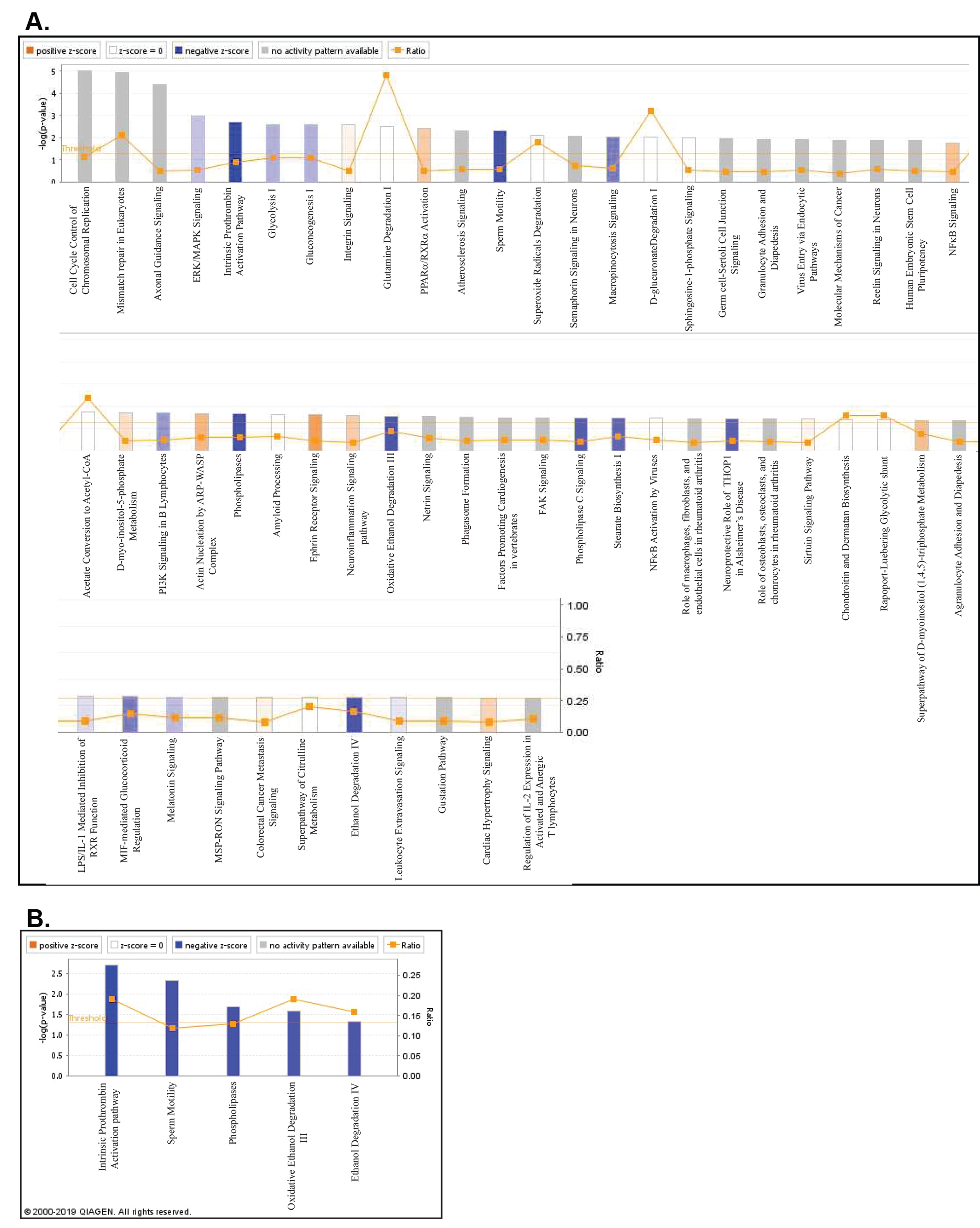

Supplement: S1 Fig — Canonical pathways associated with human genes with 2-fold expression change by RNA-seq in the AGS-EBV and Clone 1 and pB derivative tumors when compared to the AGS tumors. (A) Top canonical pathways associated with AGS-EBV tumors compared to AGS tumors. (B) Significant canonical pathways (absolute z-score ≥2) associated with AGS-EBV tumors compared to AGS tumors. The height of the bars reflects the p value, and the orange boxes reflect the ratio of the number of genes in the data set represented in the pathway. (TIF) [file ppat.1008071.s001.tif]

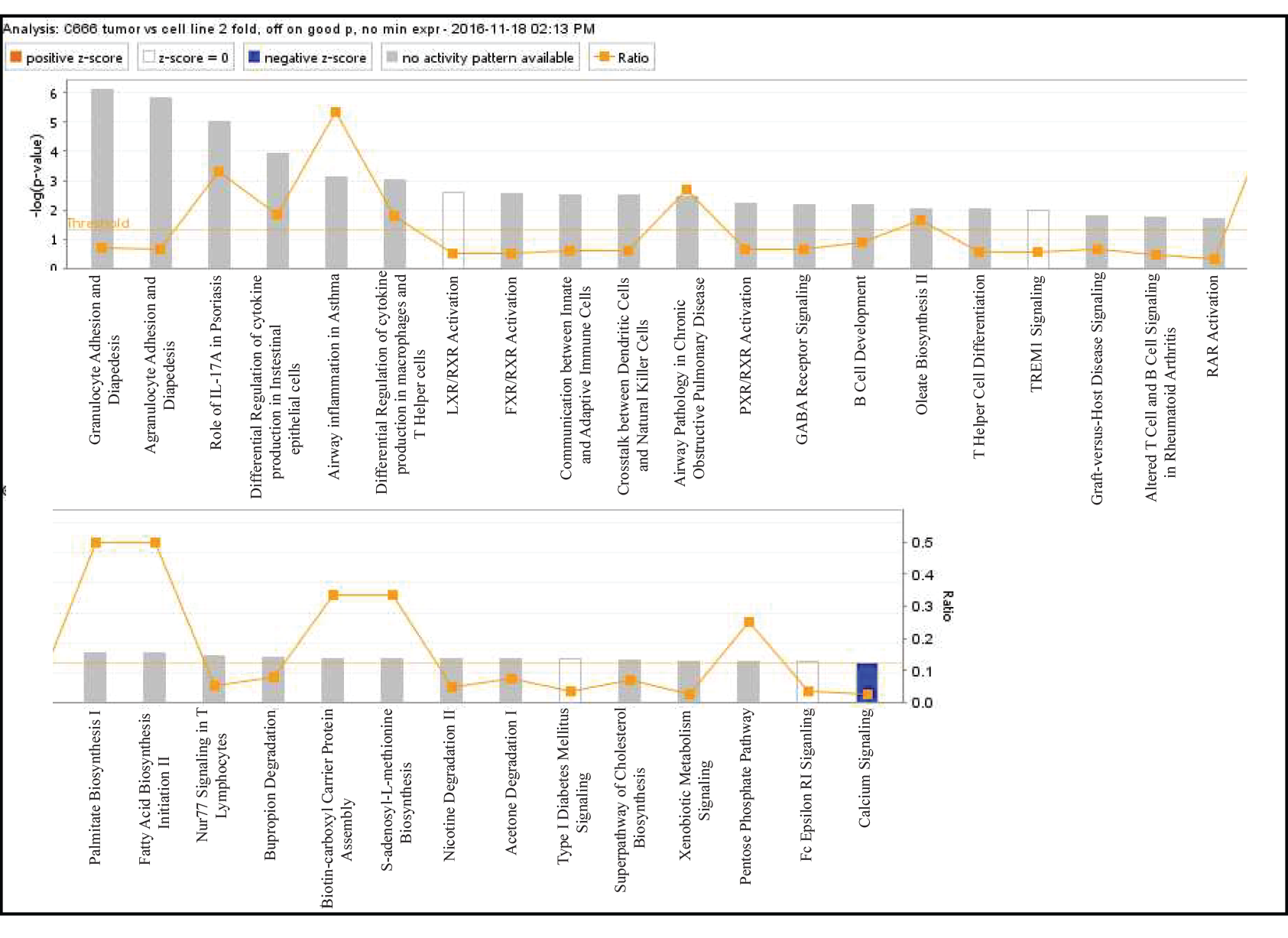

Supplement: S2 Fig — Top canonical pathways associated with human genes with 2-fold expression change by RNA-seq in the C666.1 tumors when compared to the C666.1 cell line. The height of the bars reflects the p value, and the orange boxes reflect the ratio of the number of genes in the data set that are represented in the pathway. (TIF) [file ppat.1008071.s002.tif]

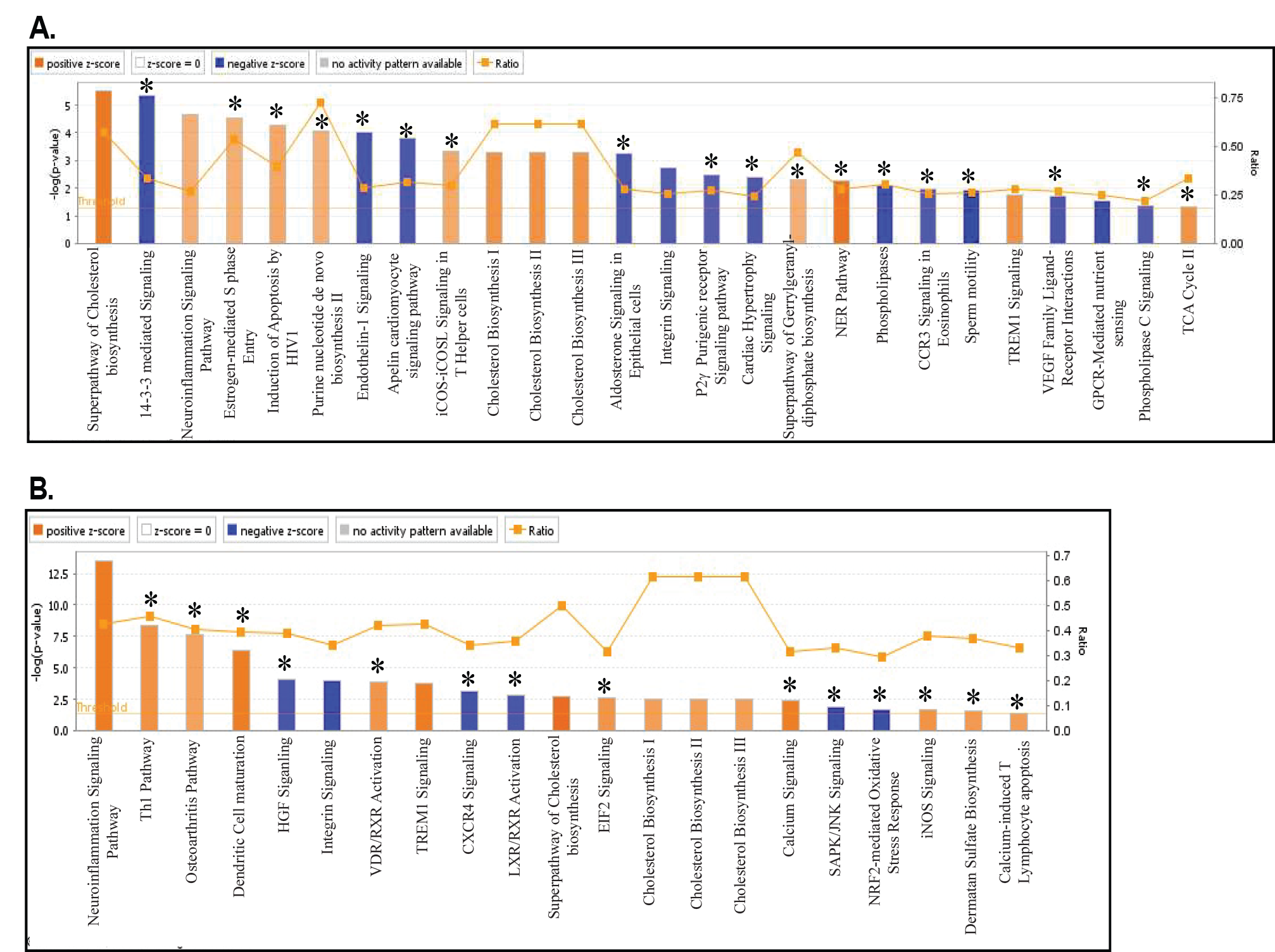

Supplement: S3 Fig — A. Significant canonical pathways predicted for the NPC tumors vs the AGS tumors. Significant canonical pathways (absolute z-score ≥2) associated with the human genes with 2-fold expression change by RNA-seq in the NPC tumors when compared to the AGS tumors. The height of the bars reflects the p value, and the orange boxes reflect the ratio of the number of genes in the data set that are represented in the pathway. Astericks (*) denote pathways unique to the comparison of NPC tumors to AGS tumors. B. Significant canonical pathways predicted for the NPC tumors vs the AGS-EBV tumors. Significant canonical pathways (absolute z-score ≥2) associated with the human genes with 2-fold expression change by RNA-seq in the NPC tumors when compared to the AGS-EBV tumors. The height of the bars reflects the p value, and the orange boxes reflect the ratio of the number of genes in the data set that are represented in the pathway. Astericks (*) denote pathways unique to the comparison of NPC tumors to AGS-EBV tumors. (TIF) [file ppat.1008071.s003.tif]

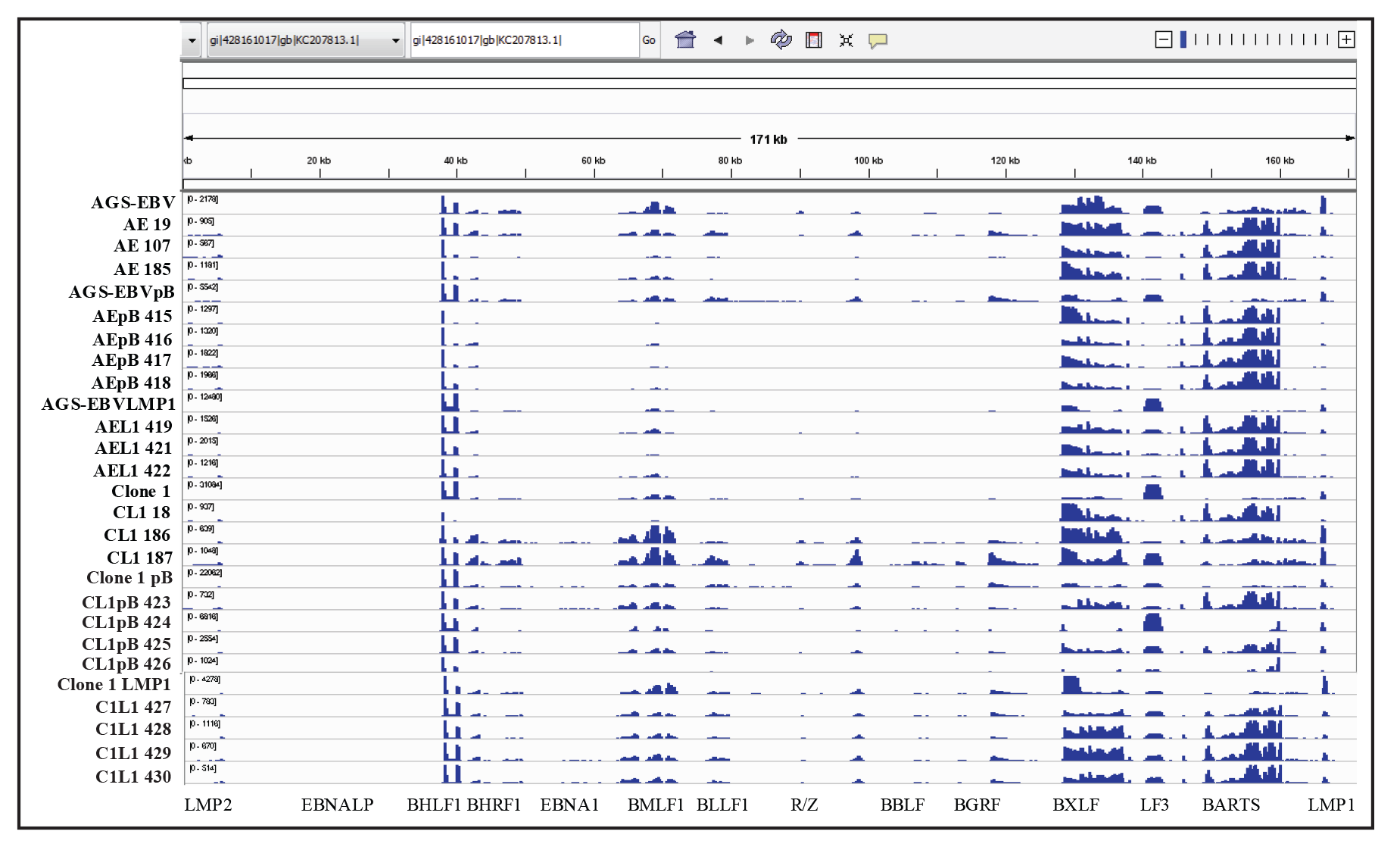

Supplement: S4 Fig — Mapped reads of AGS-EBV cell lines and tumors mapped to the Akata genome. The number of reads correlate with the height of the blue peaks. (TIF) [file ppat.1008071.s004.tif]
