# Supplementary material for: Alterations in cellular expression in EBV infected epithelial cell lines and tumors
Source: PLoS Pathog. 2019 Oct 4;15(10):e1008071. doi: 10.1371/journal.ppat.1008071 (PMC6795468; doi:10.1371/journal.ppat.1008071)
Supplement: S1 Table — (DOCX) [file ppat.1008071.s005.docx]

S1 Table. Predicted Common and Unique Upstream Regulators in Gastric tumors vs cell lines

| Common upstream regulators inhibited in both EBV^+^ and EBV^-^ tumors | Upstream regulators uniquely inhibited in EBV^+^ gastric tumors | Upstream regulators uniquely inhibited in EBV^-^ gastric tumors | Common upstream regulators activated in both EBV^+^ and EBV^-^ tumors | Upstream regulators uniquely activated in EBV^+^ gastric tumors | Upstream regulators uniquely activated in EBV^-^ gastric tumors |
| --- | --- | --- | --- | --- | --- |
| ATF4 | CDKN1A | AIF1 | COL18A1 | ALDH2 | DYRK1A |
| CARM1 | CHUK | CCND1 | miR-199a-5p (-)* | HDAC1 | HNF1A |
| CREB1 | CREM | CCNE1 | miR-30c-5p (+)* | miR-27a-3p (+)* | KEAP1 |
| EDN1 | DDIT3 | CCNK |  | miR-8 | KLF2 |
| EGFR | EGR1 | CSF2 |  | TRIM24 | let-7 (MIRLET7BHG) (3.2)** |
| FOXO1 | ELF4 | DBI |  |  | let-7a-5p |
| HIF1A | EP300 | E2F2 |  |  | miR-122 |
| KLF5 | EZH2 | E2F3 |  |  | miR-124-3p |
| MAP2K1 | F2RL1 | EIF4E |  |  | miR-133a-3p (-) |
| MAP2K1/2 | GAST | ETS1 |  |  | miR-1-3p (-) |
| MAP2K4 | IGF1R | IL15 |  |  | miR-145-5p (+) |
| MET | IPMK | KRAS |  |  | miR-15 (+) |
| PRKCE | IRS1 | MKL1 |  |  | miR-155-5p (-) |
|  | NFATC3 | MYC |  |  | miR-16-5p (+) |
|  | NOTCH1 | SREBF2 |  |  | miR-203a-3p (+) |
|  | NUPR1 | TCF7L2 |  |  | miR-21 (+) |
|  | PPRC1 |  |  |  | miR-21-5p |
|  | RHOB |  |  |  | miR-26a-5p (+) |
|  | RPS6KA5 |  |  |  | miR-291a-3p |
|  | SRF |  |  |  | miR29b-3p (+) |
|  | Stat3-Stat3 |  |  |  | miR-30a-3p (+) |
|  | TEAD1 |  |  |  | miR-34 (MIR34HG) (2.6)** |
|  | TGFA |  |  |  | miR-34a-5p |
|  | YAP1 |  |  |  | miR-503-5p (-) |
|  |  |  |  |  | miR-92a-3p (+) |
|  |  |  |  |  | TCF3 |

*known to not be expressed (-) or expressed (+) in the inoculating cell line [9]

**fold change in expression vs cell line from RNASeq data
