# Supplementary material for: Alterations in cellular expression in EBV infected epithelial cell lines and tumors
Source: PLoS Pathog. 2019 Oct 4;15(10):e1008071. doi: 10.1371/journal.ppat.1008071 (PMC6795468; doi:10.1371/journal.ppat.1008071)
Supplement: S2 Table — Shown are the genes consistently upregulated or down regulated in all the EBV+ samples with the fold expression change. (DOCX) [file ppat.1008071.s006.docx]

S2 Table. Genes changed in same direction in all EBV^+^ samples as compared to EBV^-^ samples

| Genes upregulated in all EBV^+^ samples* | ACD (2.3), ACTR1B (1.4), ACTR6 (1.7), AKR7A2 (1.5), ANKRD39 (2.1), AP1B1 (1.4), APEX2 (1.7), ARIH1 (1.6), ARL2BP (1.8), ATP6V1F (1.4), AXIN1 (1.3), BCL7B (1.5), BTBD2 (1.8), C11orf84 (3.2), CAND1 (1.6), CASP2 (1.7), CASP8AP2 (1.9), CCHCR1 (1.6), CCNT1 (1.5), CCT2 (2.0), CHERP (1.8), CHST14 (3.4), CLSPN (1.8), CMAS (1.5), COPS6 (1.4), CS (1.6), CSNK1G2 (2.0), CSTF2T (1.5), CUEDC2 (2.0), CXorf38 (1.5), DHX30 (1.7), DNA2 (1.7), DNAJC14 (1.8), DUSP14 (2.9), DYNC1LI1 (1.4), EDC3 (1.4), EDC4 (1.5), ELAC2 (1.4), EMD (1.4), EPB41L2 (1.9), EXOC3 (1.5), FAM131A (1.7), FAM49B (1.6), FLOT2 (2.5), FN3KRP (1.5), FRMD8 (1.6), FXR1 (1.7), GALK1 (3.5), GATSL2 (3.1), GDF11 (2.2), GIT1(1.6), GPSM3 (3.9), GRINA (1.6), GTF2H3 (1.7), HDGFRP2 (1.7), HPS5 (1.5), ICMT (1.6), IL10RB (1.8), ISG20L2 (1.4), KHDRBS1 (1.5), KIF2A (1.8), KPNA6 (1.5), LANCL1 (1.9), LINC00094 (2.0), LLGL1 (2.1), LMNB2 (2.5), LRRC61 (3.8), LRWD1 (2.5), MAP1S (2.2), MAP4 (1.9), MB21D1 (2.4), MCM6 (2.9), MEAF6 (1.2), METAP2 (1.5), MEX3D (1.8), MFAP1 (1.5), MGRN1 (1.5), MLLT1 (1.5), MSH6 (2.3), MTFMT (1.6), MTRF1L (1.5), MYBL1 (2.4), NAA25 (1.4), NACC1 (2.2), NCAPD3 (2.0), NCK1 (3.7), NDUFB4 (1.6), NF2 (1.8), NFE2L1 (1.8), NGRN (1.4), NIPSNAP1 (1.9), NPLOC4 (1.6), NUP62 (1.5), OAZ2 (1.7), ORAI3 (2.2), PARPBP (1.6), PEA15 (1.6), PELI3 (2.0), PHF13 (1.5), PHLPP2 (2.0), PHTF2 (2.0), PLEKHA8 (1.7), PLEKHM2 (1.6), POLDIP2 (1.5), POM121C (1.9), POMZP3 (1.7), PPP1CA (1.4), PPP1R9B (1.8), PQLC1 (2.3), PRKACA (1.8), PTBP3 (1.4), RAF1 (1.3), RARA (2.1), REPIN1 (2.4), RIC8A (1.6), RPUSD3 (1.7), RRM1 (1.8), SAMD1 (2.5), SAP130 (1.8), SEPHS1 (1.4), SLBP (1.9), SLC25A11 (1.3), SLX4 (2.0), SMARCB1 (1.5), SMARCC1 (1.7), SMARCD2 (1.4), SNAP47 (1.5), SPATS2 (1.5), ST13 (1.7), SUZ12 (1.6), SZRD1 (1.4), TBC1D24 (1.7), TBC1D25 (1.4), TCF19 (1.9), TIMM17B (1.7), TIMM8A (1.6), TK1 (2.7), TMEM106C (1.4), TMEM206 (2.3), TOR1A (1.4), TPST1 (1.9), TRAFD1 (1.7), TRIM37 (1.7), TRRAP (1.9), TSHZ1 (2.6), TTL (1.6), TTLL4 (1.9), UBAP2 (1.5), UBE2A (1.6), UBFD1 (1.9), UBL4A (1.5), UBQLN1 (1.4), UBXN4 (1.4), UCK1 (2.0), UCK2 (1.8), UNC119 (1.9), UNC45A (1.6), UPF1 (1.5), VOPP1 (1.8), WBSCR16 (1.7), XRCC5 (1.6), ZC3H18 (1.9), ZNF282 (1.5), ZNF286A (2.1), ZNF746 (1.6) |
| --- | --- |
|  |  |
| Genes down regulated in all EBV^+^ samples* | ALDH3A1 (-4.8), ALDH3A2 (-1.8), ARHGEF1 (-3.0), ATF7IP2 (-3.6), ATP6V0E1 (-1.7), ATP8A1 (-2.1), AUP1 (-1.5), BACE2 (-2.5), C14orf79 (-1.7), CAPN2 (-1.5), CAPN8 (-4.7), CEACAM5 (-8.6), CEACAM6 (-8.5), CKMT1A (-4.8), CLMN (-4.5), CTAGE5 (-3.1), CTSE (-11.0), CXCL1 (-4.2), CYSTM1 (-4.8), DGKD (-2.2), EFNB2 (-2.4), EGLN3 (-3.9), ELF3 (-2.9), FAM83E (-6.0), FGF18 (-2.2), FUCA2 (-2.3), FUT1 (-7.0), GALNT3 (-3.2), GAS6-AS1 (-12.1), GATA6 (-4.0), GMDS (-2.2), GRB14 (-3.9), IZUMO1 (-9.4), KCNE3 (-12.7), KRT6B (-5.3), LCN2 (-13.4), LGALS4 (-3.5), LIPH (-8.6), LYZ (-6.2), MAMSTR (-5.0), MECOM (-3.0), MLPH (-8.7), MSLN (-8.9), MUC1 (-11.7), MUC20 (-8.5), NTN4 (-3.5), PALD1 (-4.8), PGC (-41.0), POF1B (-5.8), POLD3 (-1.5), PPIB (-1.5), PTPRH (-13.4), RNF144B (-4.7), RPL37 (-2.1), S100P (-15.4), SCEL (-6.6), SERPINA3 (-3.7), SH3BGRL2 (-2.4), SLC9A2 (-6.9), SLPI (-12.5), SORBS2 (-3.5), STXBP2 (-1.3), TBC1D4 (-2.6), TFF1 (-20.2), TMEM181 (-1.6), TMPRSS3 (-51.7), TMSB4X (-2.8), TNFSF13 (-2.9), TPMT (-2.3), TRIM24 (-1.5), TRIM36 (-1.8), UNC13B (-1.7), VILL (-4.8), ZG16B (-2.6) |

*fold change listed is AGS-EBV tumor vs AGS tumor, but the same trend is seen in the EBV^+^ cell lines and NPC
