# Supplementary material for: Alterations in cellular expression in EBV infected epithelial cell lines and tumors
Source: PLoS Pathog. 2019 Oct 4;15(10):e1008071. doi: 10.1371/journal.ppat.1008071 (PMC6795468; doi:10.1371/journal.ppat.1008071)
Supplement: S3 Table — A. Disease and functions predicted by IPA for the 166 genes upregulated in all EBV+ samples. B. Disease and functions predicted by IP for the 74 genes down regulated in all EBV+. (DOCX) [file ppat.1008071.s007.docx]

S3 Table. Disease and Functions predicted for the 240 genes consistently changed in EBV^+^ samples

A. Disease and Functions predicted for 166 genes upregulated in all EBV^+^ samples

| Categories | Diseases or functions annotation | p-value | Predicted activation State | Genes |
| --- | --- | --- | --- | --- |
| Organismal Survival | organismal death | 0.0173 | Decreased | ACD, AXIN1, CASP2, DNA2, FXR1, GDF11, GIT1, ICMT, KHDRBS1, KIF2A, LLGL1, LMNB2, METAP2, MLLT1, MSH6, NACC1, NCK1, NF2, NFE2L1, NUP62, PEA15, PRKACA, RAF1, RARA, SLX4, SMARCB1, SMARCC1, SUZ12, TK1, TOR1A, TRAFD1, TRRAP, TSHZ1, TTL, UBE2A, UBL4A, UPF1, XRCC5 |
| Cancer, Organismal Injury and Abnormalities | incidence of malignant tumor | 0.00812 | Decreased | DNA2, MSH6, NF2, PRKACA, SMARCB1, XRCC5 |
| DNA Replication, Recombination and Repair | repair of DNA | 2.5E-07 | Increased | DNA2, GTF2H3, MSH6, MYBL1, NFE2L1, RARA, RRM1, SLX4, SMARCB1, SMARCC1, ST13, TRRAP, UBE2A, UPF1, XRCC5 |
| Infectious Diseases | Viral Infection | 0.0184 | Increased | AP1B1, CASP2, CCNT1, CCT2, CHST14, FLOT2, FXR1, IL10RB, ISG20L2, KHDRBS1, MAP1S, MAP4, MB21D1, NACA2, NF2, NUP62, PELI3, PRKACA, RAF1, RARA, REPIN1, RRM1, SMARCB1, ST13, SUZ12, TIMM17B, TIMM8A, TRAFD1 |
| Gene Expression | transactivation of RNA | 0.00050 | Increased | AXIN1, CCNT1, CCT2, CHST14, ISG20L2, KHDRBS1, MYBL1, NFE2L1, PRKACA, RAF1, RARA, SMARCB1, SMARCC1, TRRAP, XRCC5 |

B. Disease and Functions predicted for the 74 genes downregulated in all EBV^+^ samples

| Categories | Diseases or functions annotation | p-value | Predicted activation State | Genes |
| --- | --- | --- | --- | --- |
| Cell Death and Survival | cell survival | 0.00012 | Decreased | CAPN2, CEACAM5, CXCL1, EFNB2, EGLN3, ELF3, FGF18, GALNT3, GATA6, LCN2, LYZ, MECOM, NTN4, PPIB, S100P, SLPI, TNFSF13, TPMT |
| Cellular Movement | cell movement | 2.9E-07 | Decreased | ARHGEF1, ATP8A1, CAPN2, CEACAM6, CTSE, CXCL1, DGKD, EFNB2, EGLN3, ELF3, GATA6, KCNE3, KRT6B, LCN2, LYZ, MUC1, MUC20, NTN4, PPIB, PTPRH, S100P, SERPINA3, SLPI, TFF1, TMSB10/TMSB4X, TNFSF13, ZG16B |
| Cancer, Organismal Injury and Abnormalities | growth of tumor | 0.0156 | Decreased | CAPN2, CXCL1, EFNB2, FGF18, GALNT3, LCN2, MUC1, SLPI, TNFSF13 |
| Inflammatory Response | immune response of cells | 0.00528 | Decreased | CEACAM6, CXCL1, ELF3, LCN2, MUC1, TNFSF13, TRIM24, TRIM36 |
| Cancer, Growth and Proliferation | proliferation of tumor cells | 0.00521 | Decreased | CXCL1, FGF18, GALNT3, LCN2, MUC1, SLPI, TNFSF13 |
| Cardiovascular System Development and Function | migration of vascular endothelial cells | 0.00069 | Decreased | CXCL1, EFNB2, GATA6, TMSB10/TMSB4X, ZG16B |
| Cell Death and Survival | apoptosis of tumor cell lines | 0.00706 | Increased | CAPN2, CEACAM6, DGKD, GATA6, GMDS, LCN2, MECOM, MUC1, RNF144B, SERPINA3, SLPI, TMSB10/TMSB4X, TNFSF13 |
