# Supplementary material for: Alterations in cellular expression in EBV infected epithelial cell lines and tumors
Source: PLoS Pathog. 2019 Oct 4;15(10):e1008071. doi: 10.1371/journal.ppat.1008071 (PMC6795468; doi:10.1371/journal.ppat.1008071)
Supplement: S4 Table — A. List of top 100 down regulated genes in each data set and the fold change range. B. List of the top 100 upregulated genes in each data set and the fold change range. (DOCX) [file ppat.1008071.s008.docx]

S4 Table. Top 200 changed genes in each data set*

A. Top 100 down regulated genes in each data set

| AGS tumor  vs  AGS cell line  (^-^5000 to ^-^14 fold) | ACKR2, **AKR1C1**, **AKR1C2**, **ANKRD1**, APCDD1L, APCDD1L-AS1, APOBEC3B-AS1, **ARHGAP29**, **ASNS**, BDKRB1, BDKRB2, **C4BPB**, C6orf25, CALD1, CASC19, CCAT1, CD22, **CD55**, CD83, CDH11, CDH17, **CLIP4**, CNTNAP2, COL4A1, CREB5, **CTGF**, **CTH**, DUOXA1, **FLG**, FLG2, **FLG-AS1**, **FLJ22447**, GCLM, **GCNT3**, GFPT2, GPAT3, **HEG1**, HMOX1, HPGD, **HRNR**, **HSPB8**, **IGF2BP3**, KIF21B, **KLHL5**, **KLK14**, KRT16P2, KRT6A, **KRT71**, LBH, LINC00662, **LINC01300**, LINC01564, **LOC100268168**, LOC100286922, LOC100506860, LOC101927136, LOC101927501, LOC101927780, LOC101928994, LOC339166, LUCAT1, LY6G6C, LYPD6B, MALL, NAV3, NCF2, NKAIN4, NLRP7, **NNMT**, NPPB, NRP1, OLR1, PCED1B, PDGFRA, **PHLDB2**, PSAT1, **PSG1**, **PSG11**, **SAMD4A**, SCARNA26A, SERPINB2, SGTB, SLC2A3, **SLC7A11**, SLC7A7, SPANXN3, **SPRR2D**, SRGAP2C, SYTL3, **TENM3**, TLR6, TMEM52B, TNNT1, TRABD2B, TSPAN2, TXNRD1, **UCA1**, VANGL2, ZBED2, **ZNF114** |
| --- | --- |
| AGS-EBV tumor  vs  AGS-EBV cell line  (^-^234 to ^-^9 fold) | ADGRA2, **AKR1C1**, **AKR1C2**, AKR1C3, **ANKRD1**, **ARHGAP29**, **ASNS**, BEST1, **C4BPB**, CBS, **CD55**, CHAC1, **CLIP4**, CST4, **CTGF**, **CTH**, CYP1A1, DDR2, DEFB1, DHRS2, DLC1, ECM2, EGR1, EPAS1, ESM1, FGF21, **FLG**, **FLG-AS1**, **FLJ22447**, GAST, **GCNT3**, GDF15, GLI1, GPR1, GZMB, **HEG1**, **HRNR**, **HSPB8**, ID3, **IGF2BP3**, IL20RB, INHBE, KLF9, KLHDC7B, **KLHL5**, **KLK14**, **KRT71**, KRT86, KRTAP3-1, LINC01133, **LINC01300**, LINC01356, **LOC100268168**, LOC100996286, MAP1B, MEIS3, MEIS3P1, MOB3B, MYH13, MYH4, MYHAS, MYL7, NID1, **NNMT**, NOS2, NRCAM, PER1, **PHLDB2**, PIM1, PIP5KL1, PLAC1, PRPH, PRSS35, PSAPL1, **PSG1**, **PSG11**, PSG2, PSG6, PTGS2, RTN4RL2, S100P, SALL4, **SAMD4A**, SBSN, SEMA5A, SIPA1L2, SLC43A1, **SLC7A11**, SLC7A11-AS1, SLMO2-ATP5E, **SPRR2D**, ST6GALNAC3, SULT1C4, **TENM3**, TMEM156, TRIB3, **UCA1**, UNC5B, UPK2, **ZNF114** |
| AGS-EBV cell line  vs  AGS cell line  (^-^388 to ^-^14 fold) | AIF1, AKR1B10, AKR1C1, AKR1C8P, APCDD1L-AS1, APOBEC3B-AS1, **ASS1**, BDKRB2, C12orf40, C1orf61, C6orf223, CD22, CDH11, CDH2, CEACAM6, CELA3A, CES5A, CLIC5, CNTNAP3P2, COX8C, CSF2RB, CYP4F11, DEFB103A, DEFB103B, DENND2C, DMRTA1, DPYSL3, EBLN1, ELSPBP1, FABP1, FAM155A, FAM27C, FLJ22447, **GALNT5**, GFPT2, GPX2, HMOX1, HTR3A, HTRA1, HTRA3, IL1B, IQCF5, JDP2, KRT13, KRT17, KRT6A, LARGE-AS1, LCE1F, LHFPL5, LILRB1, LINC00341, LINC01364, LINC01554, LINC01559, LINC01564, LINC01629, LOC100131107, LOC100287225, LOC100507431, LOC101927780, LOC101928861, LOC101928994, LOC102724301, LOC102724450, LOC105378127, LOC339166, LRTM2, LVCAT8, MAGEA2, MAGEB5, MAGEC2, MMP2, NKAIN4, NXPE4, OLR1, PHACTR3, PI3, PLCE1-AS2, PMP2, POF1B, PRR16, PYGL, REG4, SCARNA13, SCEL, SERPINB2, SPANXN3, SPATA31A5, SPATA31A7, SPINK7, SULT1A2, SUN3, SYNE3, TDRD9, TMEM52B, TREML3P, TSPAN2, ZBED2, ZNF280A, ZXDA |
| AGS-EBV tumor  vs  AGS tumor  (^-^886 to ^-^12 fold) | ACSS1, ADGRF1, ADIRF, AFAP1-AS1, ALG1L, ALOX5, APH1B, AQP5, **ASS1**, ATP13A4, AVIL, BATF, BCAS1, BMP7, C11orf53, C2orf70, CAPN12, CATSPERB, CD27-AS1, CDC42EP3, CHST15, CLCNKB, CLDN18, COL11A2, COL9A3, CTSS, CXCL17, DBN1, DFNB31, DPCR1, ELF5, ELFN1-AS1, ENO2, FAM69C, FBP2, FES, FOXI1, FUT3, GAL3ST1, **GALNT5**, GAS6-AS1, GATA5, GOLT1A, GPSM1, GSDMD, GYG2, H2BFWT, HCG4B, HEPACAM2, HRASLS5, KIAA1211L, KLK10, KLK11, KLK7, LCN2, LINC00935, LINC01310, LOC101928738, LOC102724156, LOC284023, LPAR6, LRMP, MCAM, MIR3142HG, MMP23A, MRC2, NRSN2, NYNRIN, PALM, PAX8-AS1, PDE9A, PDIA2, PGC, PLA2G2A, PMEPA1, PROC, RASIP1, RNF183, SCNN1G, SECTM1, SEMA3F, SLC17A9, SLC44A4, SLC9A4, SOD3, SOX10, SPINK5, SPON2, TFF1, TFF2, TMPRSS3, TNFAIP2, TNFRSF14, TNFSF12, TP53I11, TPM2, UGT1A10, VSIG1, VSIG2, WNT11 |

B. Top 100 upregulated genes in each data set

| AGS tumor  vs  AGS cell line  (5000 to 80 fold) | AATK, ACSS1, ACY3, AGR2, ANKRD22, APH1B, **ARHGEF19**, ATP13A4, BANF2, BMP7, BPIFB1, C11orf21, C11orf53, C2orf70, CA9, CAPN3, CATSPERB, CDC42EP3, **CFI**, CLDN18, COL9A3, CTSS, CXCL17, **CXCR4**, CYP2B7P, DAPK1, **DAPL1**, DFNB31, DPCR1, ELF5, **EVL**, FBP1, FBP2, FCGBP, FES, FMO5, FSBP, GAL3ST1, GATA5, **GJB1**, GLOD5, GLYATL1, GOLT1A, GYG2, H2BFWT, HEPACAM2, HOXA3, KIAA1211L, KLF3-AS1, KLK11, LGALS2, LINC00238, **LINC00675**, LINC00896, LINC00935, LINC01310, LINC01473, LOC101927196, LOC101927604, LOC102724156, LOC283710, LOC730102, **LYZ**, MCAM, MIR3142HG, MRC2, MUC6, PAX8-AS1, PDZD3, PGC, PIGR, PLEKHS1, PTGER2, **RHBDL2**, **RNASE1**, SCNN1G, SEC16B, SEMA4A, SGK2, SHISA4, SLC44A4, SLC9A3, SLC9A4, SNX29P2, SOX10, **SPDEF**, SPNS2, **SRGN**, STARD13, TACR2, **TBXAS1**, TFF2, TFF3, TNFSF12, TRPM3, TSPAN32, UBA7, UGT1A10, VSIG1, VSIG2 |
| --- | --- |
| AGS-EBV tumor  vs  AGS-EBV cell line  (978 to 28 fold) | ADAMTS6, ADAMTSL2, AOC1, APCDD1, APOA1, APOBEC3G, APOH, AQP2, **ARHGEF19**, AZGP1, C2orf82, C6orf223, CA6, CACNA1E, CCL24, CEMIP, **CFI**, CHADL, CLDN2, COLGALT2, CRYBB1, CTLA4, **CXCR4**, DACT1, **DAPL1**, DBH-AS1, DDC, DKK4, DPEP1, ENTPD8, **EVL**, EXOC3L2, FAIM2, FAM3B, FGF3, FHL1, FYN, GIMAP2, **GJB1**, GMPR, GZMA, HMGCS2, HNF1A-AS1, HSD17B2, IHH, ITIH6, JAKMIP2-AS1, KIAA1217, LEF1, LGALS12, LGALS9, **LINC00675**, LINC01512, LOC100507006, **LYZ**, MAOB, MIA, MPP1, MUCL1, NFIA-AS2, NXF3, OSBPL6, P2RX1, PAGE1, PAGE4, PCDHB16, PHGR1, PNLIPRP2, POU2AF1, PPP1R1B, PPP2R2C, PRR26, PRSS33, PRSS56, PTPRO, RASGRF1, RASSF6, RGL1, **RHBDL2**, **RNASE1**, RNASE6, ROPN1, SCML4, SELENBP1, SH2D7, SLC13A2, SLC14A1, SLC39A5, SLCO2A1, SMPX, SPACA3, **SPDEF**, SPI1, **SRGN**, STRA6, **TBXAS1**, UBD, UST, ZAP70, ZG16B |
| AGS-EBV cell line  vs  AGS cell line  (151 to 2 fold) | ADGRA2, ADGRL1, ARHGEF2, ARRDC4, AXIN1, BCL6, BMF, BRD4, CBX6, CENPBD1P1, CHERP, CNOT3, CNTNAP1, COL6A3, CSNK1A1L, DHRS2, DHX30, DNAJA3, DOT1L, DUSP1, EXOSC5, FCHO1, FGD3, FOS, GIT1, GLIS2, GMEB2, GNAZ, H1F0, H1FX, **H3F3C**, HCFC1, ICOSLG, JADE2, KATNB1, KDM6B, KIAA0355, KLHL15, LINC01123, LLGL1, LOC389641, LOC407835, LONP1, LRRC4B, MAP1S, MAP2K7, MKNK2, MYH13, NACC1, NEFH, NOC2LP2, PCDHGA11, PCDHGA12, PHF21A, PHF8, PKN1, PLEKHM2, POLR2E, POMGNT2, PPP1R15A, PPP1R9B, PSAPL1, R3HDM2, RASA4, RASD1, RCAN1, RGPD8, RGS16, RNF138P1, RPL12, **RPL19P12**, RPL28, RPS27, RTN4RL2, SAFB2, SAMD1, SBF1, SF3A2, SLC2A4, SNAP47, SNCG, SNORA4, SNORA81, **SNORD141A**, **SNORD141B**, SOCS7, STK11, STRN4, SYDE2, TCF3, TLE3, TMCC2, TMEM245, TONSL, TRAP1, TRIM28, VPS37C, XYLT2, ZBTB7B, ZXDC |
| AGS-EBV tumor  vs  AGS tumor  (1000 to 4 fold) | AATF, AKR7A2P1, AQP1, ASIC1, BAMBI, C17orf82, CABP1, CACHD1, CACNA1E, CCDC148, CD52, CDK5R1, CEMIP, CPLX2, CRABP2, CRAT, CRYBB1, CTLA4, CXADRP2, ETV1, FAIM2, FAM171A2, FAM225A, FAM225B, FLJ44635, FRG2, FUT7, FYN, GALK1, GAS7, GPLD1, GPR157, GPX5, **H3F3C**, HSP90B2P, HSPA12A, IL17RD, IL27RA, ITGA1, ITPKB, KITLG, LCK, LHX1, LINC00176, LOC100129940, LOC100132111, LOC101243545, LOC644762, LRP8, MANEAL, MIR5047, MIR622, MIR6723, MNX1-AS1, MORC4, MTCL1, MUCL1, MYO3B, NACA2, NFIA-AS2, OLFML3, PAGE4, PANDAR, PCBP2-OT1, PGAM2, PNLIPRP2, PPIAL4G, PRSS56, PSMG3-AS1, RDH5, RGL1, RIPPLY1, **RPL19P12**, S100A3, SELL, SEMA3C, SEMA6B, SGTB, SLC13A2, SLC14A1, SLC1A5, SLC39A5, **SNORD141A**, **SNORD141B**, SP5, SPI1, SRCIN1, TAC3, TIAM1, TLR4, TPK1, TPST2, TTC39B, TUBB3, UBR5-AS1, UQCRBP1, VSIG10L, WTH3DI, ZDHHC8P1, ZNF697 |

*bolded font denotes genes commonly downregulated or upregulated in the dataset on Venn diagrams
