# Supplementary material for: Alterations in cellular expression in EBV infected epithelial cell lines and tumors
Source: PLoS Pathog. 2019 Oct 4;15(10):e1008071. doi: 10.1371/journal.ppat.1008071 (PMC6795468; doi:10.1371/journal.ppat.1008071)
Supplement: S5 Table — A. Disease and functions of the top 100 upregulated genes in the AGS-EBV cell lines compared the AGS cell line. B. Disease and functions of the top 100 upregulated genes in the AGS-EBV tumors compared to the AGS tumors. (DOCX) [file ppat.1008071.s009.docx]

S5 Table. Disease and functions of the top 100 up regulated genes in the AGS-EBV cell lines and tumors.

A. Disease and functions of top 100 up regulated genes AGS-EBV cell line vs AGS cell line*

| Categories | Diseases or Functions Annotation | p-value | Predicted Activation State | Activation z-score | Molecules |
| --- | --- | --- | --- | --- | --- |
| Cell Death and Survival | Cell viability | 1.25E-04 | Increased | 2.824 | **BCL6, BRD4, DOT1L, DUSP1, FOS, GIT1, ICOSLG, KDM6B, NACC1, NEFH, PCDHGA11, PCDHGA12, PKN1, RCAN1, RGS16, SBF1, SLC2A4, STK11, TCF3, TONSL, TRIM28** |
| Cellular Assembly and Organization, Cellular Function and Maintenance | Organization of cytoplasm | 7.57E-03 | Increased | 2.682 | **ADGRA2, ARHGEF2, AXIN1, BCL6, CNTNAP1, DNAJA3, FGD3, FOS, GIT1, HCFC1, KATNB1, LLGL1, LONP1,** MAP1S**, NEFH, PLEKHM2, PPP1R9B, STK11** |
| Cell-mediated Immune Response, Hematological System Development and Function | T cell homeostasis | 1.48E-02 | Increased | 2.488 | **BCL6, BMF, DNAJA3, ICOSLG, LONP1, STK11, TCF3, ZBTB7B** |
| Cellular Growth and Proliferation, Hematopoiesis | Lymphopoiesis | 6.33E-03 | Increased | 2.369 | **BCL6, CNOT3, DNAJA3, FOS, GIT1, ICOSLG, LONP1, STK11, TCF3, ZBTB7B** |
| Organismal Survival | Morbidity or mortality | 2.57E-05 | Decreased | -5.071 | **ADGRA2, AXIN1, BCL6, BMF, CNOT3, CNTNAP1, DNAJA3, DOT1L, DUSP1, FOS, GIT1, GLIS2, GNAZ, H1F0,** KDM6B, LLGL1**, MAP2K7,** NACC1**, PCDHGA11, PCDHGA12, PHF21A, POMGNT2, PPP1R15A, RCAN1,** RGPD8**, SBF1, SLC2A4, SNCG, SOCS7, STK11, TCF3, TRIM28** |

*bolded genes denote genes uniquely upregulated in the cell line to cell line comparison relative to the AGS-EBV tumor vs AGS tumor comparison (the top 100 upregulated genes in the cell line to cell line comparison are compared to the 2 fold upregulated genes in the tumor to tumor comparison)

B. Disease and functions of top 100 up regulated genes AGS-EBV tumor vs AGS tumor*

| Categories | Diseases or Functions Annotation | p-value | Predicted Activation State | Activation z-score | Molecules |
| --- | --- | --- | --- | --- | --- |
| Cellular Movement | Migration of cells | 7.19E-04 | Increased | 3.502 | **AQP1, ASIC1, CDK5R1, CEMIP, CTLA4, ETV1, FAIM2, FUT7, FYN, GPLD1, IL27RA, ITGA1, KITLG, LCK, LHX1, LRP8, PANDAR, SELL, SEMA3C, SPI1, SRCIN1, TIAM1, TLR4** |
| Cellular Movement | Cell movement | 6.21E-04 | Increased | 3.35 | **AQP1, ASIC1, CACNA1E, CDK5R1, CEMIP, CTLA4, ETV1, FAIM2, FUT7, FYN, GPLD1, IL27RA, ITGA1, KITLG, LCK, LHX1, LRP8, PANDAR, SELL, SEMA3C, SPI1, SRCIN1, TIAM1, TLR4**, TPST2 |
| Cell-To-Cell Signaling and Interaction, Immune Cell Trafficking | Adhesion of immune cells | 1.68E-03 | Increased | 2.58 | **FUT7, FYN, KITLG, LCK, SELL, SPI1, TLR4** |
| Amino Acid Metabolism, Post-Translational Modification | Phosphorylation of L-amino acid | 3.61E-05 | Increased | 2.201 | **CDK5R1, FYN, KITLG, LCK, LRP8, MYO3B, TLR4** |
| Amino Acid Metabolism, Post-Translational Modification | Phosphorylation of L-tyrosine | 5.65E-04 | Increased | 2.201 | **FYN, KITLG, LCK, LRP8, TLR4** |
| Cancer,Organismal Injury and Abnormalities | Advanced malignant tumor | 1.39E-03 | Increased | 2 | **AQP1, CD52, CEMIP, CRABP2, CTLA4, ETV1, FUT7, FYN, LCK, LRP8, OLFML3, SELL, SEMA3C, TUBB3** |

*bolded genes denote genes uniquely upregulated in the tumor to tumor comparison relative to the AGS-EBV cell line vs AGS cell line comparison (the top 100 upregulated genes in the tumor to tumor comparison are compared to the 2 fold upregulated genes in the cell line to cell line comparison)
