# Supplementary material for: Alterations in cellular expression in EBV infected epithelial cell lines and tumors
Source: PLoS Pathog. 2019 Oct 4;15(10):e1008071. doi: 10.1371/journal.ppat.1008071 (PMC6795468; doi:10.1371/journal.ppat.1008071)
Supplement: S6 Table — List of genes changed at least 1.5 fold in the EBV+ cell lines, EBV+ tumors and the BART cell line [11] when compared to the EBV- control. P values and fold changes are denoted. (DOCX) [file ppat.1008071.s010.docx]

S6 Table. Correlation with potential BARTlnc targets

| Gene | p-value  (EBV^+^ tumor vs. EBV^-^ tumor ) | Fold change (EBV^+^ tumor vs. EBV^-^ tumor ) | p-value (BART cell line vs vector control )* | Fold change (BART cell line vs vector control)* | Name |
| --- | --- | --- | --- | --- | --- |
| TMPRSS3 | 1.83E-22 | -51.74 | 5.35E-05 | -3.33 | Transmembrane Protease, Serine 3 |
| TFF1 | 9.09E-09 | -20.18 | 1.22E-06 | -4.18 | Trefoil Factor 1 |
| PTPRH | 2.03E-10 | -13.45 | 3.47E-07 | -3.90 | Protein Tyrosine Phosphatase, Receptor Type H |
| LCN2 | 4.11E-09 | -13.42 | 1.45E-04 | -45.56 | Lipocalin 2 |
| SLPI | 1.45E-18 | -12.51 | 1.77E-03 | -1.83 | Secretory Leukocyte Peptidase Inhibitor |
| MUC1 | 6.67E-11 | -11.73 | 4.15E-07 | -1.86 | Mucin 1 |
| MSLN | 4.57E-16 | -8.93 | 3.46E-05 | -1.50 | Mesothelin |
| CEACAM5 | 5.24E-04 | -8.57 | 2.67E-08 | -2.23 | Carcinoembryonic Antigen Related Cell Adhesion Molecule 5 |
| FUT1 | 2.92E-11 | -6.97 | 1.81E-05 | -3.29 | Fucosyltransferase 1 |
| FAM83E | 1.25E-09 | -5.95 | 2.81E-03 | -2.09 | Family With Sequence Similarity 83 Member E |
| MAMSTR | 5.83E-07 | -4.96 | 4.11E-03 | -1.62 | MEF2 Activating Motif And SAP Domain Containing Transcriptional Regulator |
| PALD1 | 3.87E-06 | -4.81 | 1.01E-02 | -1.76 | Phosphatase Domain Containing, Paladin 1 |
| RNF144B | 9.55E-09 | -4.74 | 1.10E-03 | -2.15 | Ring Finger Protein 144B |
| CXCL1 | 1.95E-03 | -4.24 | 2.51E-03 | -2.76 | C-X-C Motif Chemokine Ligand 1 |
| SERPINA3 | 4.41E-03 | -3.68 | 8.05E-05 | -6.28 | Serpin Family A Member 3 |
| NTN4 | 3.03E-05 | -3.49 | 1.98E-08 | -3.18 | Netrin 4 |
| GALNT3 | 5.76E-09 | -3.20 | 1.44E-05 | -1.97 | Polypeptide N-Acetylgalactosaminyltransferase 3 |
| CTAGE5 | 1.75E-16 | -3.12 | 5.72E-04 | -1.72 | CTAGE Family Member 5, ER Export Factor |
| SH3BGRL2 | 2.65E-06 | -2.40 | 1.91E-05 | -1.70 | SH3 Domain Binding Glutamate Rich Protein Like 2 |
| DGKD | 7.13E-08 | -2.16 | 2.93E-05 | -1.80 | Diacylglycerol Kinase Delta |
| TRIM36 | 1.77E-06 | -1.83 | 4.38E-04 | -2.12 | Tripartite Motif Containing 36 |
|  |  |  |  |  |  |
| MPP2 | 2.6E-02 | 11.3 | 7.00E-04 | 2.08 | Membrane Palmitoylated Protein 2 |
| SNRNP25 | 3.5E-02 | 132 | 8.37E-05 | 1.67 | Small Nuclear Ribonucleoprotein U11/U12 Subunit 25 |
| S1PR2 | 2.3E-04 | 1.57 | 1.70E-03 | 17.77 | Shingosine-1-Phosphate Receptor 2 |
| TSPAN9 | 7.0E-04 | 1.55 | 1.05E-02 | 39.04 | Tetraspanin 9 |
| UNC119 | 2.76E-04 | 1.86 | 5.25E-05 | 1.67 | Unc-119 Lipid Binding Chaperone |
| PELI3 | 2.04E-04 | 2.02 | 2.32E-03 | 1.69 | Pellino E3 Ubiquitin Protein Ligase Family Member 3 |

*data derived from [11]
